# Effect of Homocysteine-Lowering Nutrients on Blood Lipids: Results from Four Randomised, Placebo-Controlled Studies in Healthy Humans

**DOI:** 10.1371/journal.pmed.0020135

**Published:** 2005-05-31

**Authors:** Margreet R Olthof, Trinette van Vliet, Petra Verhoef, Peter L Zock, Martijn B Katan

**Affiliations:** **1**Wageningen Centre for Food Sciences and Division of Human Nutrition, Wageningen UniversityWageningenthe Netherlands; **2**TNO Quality of LifeZeistthe Netherlands; Children's Hospital BostonUnited States of America

## Abstract

**Background:**

Betaine (trimethylglycine) lowers plasma homocysteine, a possible risk factor for cardiovascular disease. However, studies in renal patients and in obese individuals who are on a weight-loss diet suggest that betaine supplementation raises blood cholesterol; data in healthy individuals are lacking. Such an effect on cholesterol would counteract any favourable effect on homocysteine. We therefore investigated the effect of betaine, of its precursor choline in the form of phosphatidylcholine, and of the classical homocysteine-lowering vitamin folic acid on blood lipid concentrations in healthy humans.

**Methods and Findings:**

We measured blood lipids in four placebo-controlled, randomised intervention studies that examined the effect of betaine (three studies, *n* = 151), folic acid (two studies, *n* = 75), and phosphatidylcholine (one study, *n* = 26) on plasma homocysteine concentrations. We combined blood lipid data from the individual studies and calculated a weighted mean change in blood lipid concentrations relative to placebo. Betaine supplementation (6 g/d) for 6 wk increased blood LDL cholesterol concentrations by 0.36 mmol/l (95% confidence interval: 0.25–0.46), and triacylglycerol concentrations by 0.14 mmol/l (0.04–0.23) relative to placebo. The ratio of total to HDL cholesterol increased by 0.23 (0.14–0.32). Concentrations of HDL cholesterol were not affected. Doses of betaine lower than 6 g/d also raised LDL cholesterol, but these changes were not statistically significant. Further, the effect of betaine on LDL cholesterol was already evident after 2 wk of intervention. Phosphatidylcholine supplementation (providing approximately 2.6 g/d of choline) for 2 wk increased triacylglycerol concentrations by 0.14 mmol/l (0.06–0.21), but did not affect cholesterol concentrations. Folic acid supplementation (0.8 mg/d) had no effect on lipid concentrations.

**Conclusions:**

Betaine supplementation increased blood LDL cholesterol and triacylglycerol concentrations in healthy humans, which agrees with the limited previous data. The adverse effects on blood lipids may undo the potential benefits for cardiovascular health of betaine supplementation through homocysteine lowering. In our study phosphatidylcholine supplementation slightly increased triacylglycerol concentrations in healthy humans. Previous studies of phosphatidylcholine and blood lipids showed no clear effect. Thus the effect of phosphatidylcholine supplementation on blood lipids remains inconclusive, but is probably not large.

Folic acid supplementation does not seem to affect blood lipids and therefore remains the preferred treatment for lowering of blood homocysteine concentrations.

## Abstract 2

Lowering homocysteine might reduce the risk for heart disease. Betaine seems to have an adverse effect on blood lipids. This would make it less suitable than folic acid, which does not affect blood lipids.

## Introduction

Cardiovascular disease (CVD) is a major cause of morbidity and mortality in Western societies. Diet may play a crucial role in the aetiology of CVD. However, clinical trials that test the effects of diet on CVD are complicated to conduct. Hence, risk factors that are predictive of CVD risk are frequently used to test the effects of diet on disease prevention. Examples of such risk factors are homocysteine concentrations and cholesterol concentrations. A limitation of studying effects of dietary changes on risk factors is that often a single factor is measured, whereas multiple factors might be affected by changes in diet. Whether homocysteine lowering indeed lowers risk of CVD is not yet established, but evidence for a causal relationship is accumulating [1–3]. The effects of increased blood low-density lipoprotein (LDL) cholesterol concentrations on CVD risk are well established.

Betaine is used as therapy to lower plasma homocysteine in hyperhomocysteinemic patients with genetic defects in their homocysteine metabolism who are unresponsive to pyridoxine, folic acid, and vitamin B12 [4–6]. It also lowers plasma homocysteine in healthy humans with homocysteine concentrations in the normal range [7–9]. Betaine occurs naturally in the diet, or it can be produced endogenously through oxidation of choline. Dietary intake of betaine is estimated at 0.5–2 g/d generally, and major food sources are wheat products (e.g., bread), beets, and spinach [10]. Recently we showed that supplementation with 1.5 g/d of betaine, which is in the range of daily intake, lowers fasting and post-methionine plasma homocysteine concentrations in healthy humans [8]. The homocysteine-lowering properties of betaine would predict a decrease in risk of CVD. However, betaine and its precursor choline are also involved in lipid metabolism. In animal studies betaine supplementation increases serum cholesterol concentrations relative to control [11,12]. Studies in humans have also found that betaine supplementation increases serum cholesterol in obese individuals who are on a weight-loss diet, and in patients with chronic renal failure [9,13]. However, in these studies betaine was not the only intervention, so the observed effects on lipid concentrations could have been due to factors other than betaine supplementation. Furthermore, in the study with obese individuals, weight changes might have contributed to the changes in serum lipids [9]. The effects of betaine on lipid metabolism in healthy humans are not well established.

Choline, the precursor of betaine, also occurs naturally in foods, mainly as phosphatidylcholine. Daily intake of choline is generally estimated to be 0.3–1 g [14]. Phosphatidylcholine is an essential component of very low density lipoproteins (VLDLs) and is therefore involved in lipid metabolism. Animal and human studies indicate that choline deficiency decreases serum cholesterol, which can be prevented by addition of choline [15–17]. Data on the effects of choline supplementation on blood lipids in humans are scarce.

**Figure 1:**
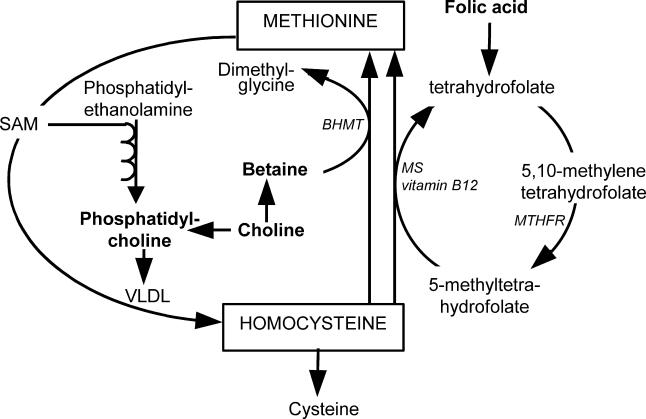
Role of Folic Acid, Choline, and Betaine in Homocysteine Metabolism and in Phosphatidylcholine Metabolism Phosphatidylcholine is necessary for synthesis of VLDL, which exports lipids from the liver.

Increases in blood lipid concentrations due to betaine or choline supplementation might offset the potential beneficial effects of their homocysteine-lowering properties. Folic acid is the most commonly used homocysteine-lowering agent. The betaine remethylation and folic acid remethylation pathways are interrelated (Figure 1), so effects of folic acid supplementation on lipid metabolism cannot be excluded [18].

In this study we investigated the effects of supplementation of three homocysteine-lowering nutrients, betaine, choline (as phosphatidylcholine), and folic acid, on blood lipid concentrations in healthy humans. For this, we combined data of four placebo-controlled studies in healthy humans, performed by our group. These are the only available randomized, controlled studies in healthy humans on this matter that we are aware of.

## Methods

Data were derived from four placebo-controlled intervention studies (studies 1, 2, 3 and 4) into the effects of betaine, phosphatidylcholine, and folic acid on homocysteine concentrations (Table 1). All studies are reported in accordance with the CONSORT guidelines (Tables S1–S4).

**Table 1:**
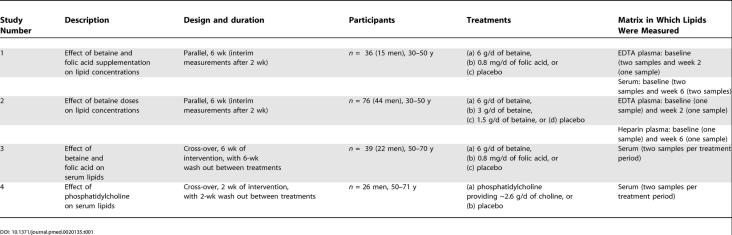
Overview of Study Designs into the Effects of Betaine, Folic Acid, and Phosphatidylcholine on Blood Lipid Concentrations

### Study 1

#### Study participants and design

**Figure 2:**
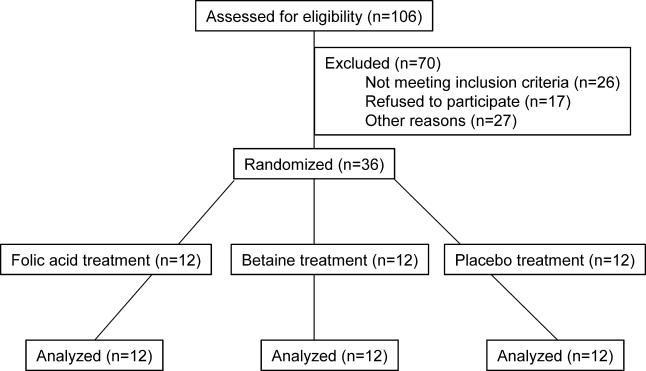
Flow Diagram of Participant Progress through Study 1

The primary endpoint of this study was plasma homocysteine concentrations, and these results have been published [7]. Blood lipid measurements were done post-hoc. Study 1 was conducted according to Good Clinical Practice guidelines at TNO Quality of Life (Zeist, the Netherlands). The local medical ethics committee approved the protocol, and all volunteers gave their written informed consent. Volunteers were recruited from April to May 2000. Eligible volunteers were healthy as assessed by routine medical screening and a general health questionnaire, had plasma total homocysteine concentrations below 25 μmol/l, had no history of cardiovascular disease, and had not used vitamin B supplements more than once a week in the 3 mo before entering the study. Out of the eligible participants, the 36 participants (15 males and 21 females, age 30–50 y) with the highest plasma total homocysteine concentrations (range 8.9 to 21.0 μmol/l) were included in this placebo-controlled, double-blind parallel study (Figure 2). Participants were stratified by gender and plasma homocysteine concentration, and then randomly assigned to one of three treatments for 6 wk: (a) 6 g/d of betaine (*n* = 12) (BUFA Pharmaceutical Products, Uitgeest, the Netherlands), (b) 0.8 mg/d of folic acid (*n* = 12), or (c) placebo (*n* = 12). One person at the local pharmacy assigned codes to the study treatments and provided the key in a sealed envelope to the principal investigator at TNO. The statistician then randomly allocated treatment codes to participant numbers. Randomization was done using a computerized procedure that produced combinations based on random seed numbers. The statistician supplied the medical investigator with sealed envelopes with the treatment allocation per participant. The participants and all others involved in this study were unaware of treatment allocation. The principal investigator performed unblinding of the treatment allocation after the study had ended, laboratory analyses were complete, and datasets were locked. The study supplements were dissolved in water and ingested on two daily occasions, one half of the daily dose after breakfast and the other half after the evening meal. Throughout the study, participants were asked to refrain from eating products based on animal liver and to consume no more than two eggs per week. (Eggs and liver are major sources of betaine and of choline, the dietary precursor for betaine.)

#### Blood sampling and blood lipid analysis

Venous blood was taken from an anticubital vein in the forearm following an overnight fast at baseline and after 2 wk and 6 wk of intervention. Immediately after collection, blood was mixed well and put on ice. Within 30 min samples were centrifuged for 10 min at 2,000 *g* at 4 °C. Samples were stored below −20 °C. Samples were coded to hide the identity and treatment of participants. All samples obtained from one participant were analysed in the same run. We measured blood lipid concentrations in fasting EDTA plasma samples at baseline and after 2 wk of intervention, and in fasting serum samples collected at baseline and after 6 wk of intervention. Concentrations of total cholesterol, high-density lipoprotein (HDL) cholesterol, and triacylglycerol were measured with a Hitachi (Tokyo, Japan) 911 analyzer and enzymatic assays of Roche (Basel, Switzerland). LDL cholesterol concentrations were calculated with the formula of Friedewald et al. [19].

### Study 2

#### Participants and design

**Figure 3:**
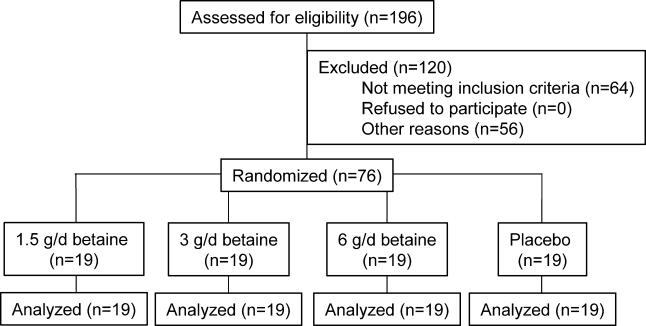
Flow Diagram of Participant Progress through Study 2

The primary endpoint of this study was plasma homocysteine concentrations, and these results have been published [8]. Blood lipid measurements were done post-hoc. Study 2 was conducted according to Good Clinical Practice guidelines at TNO Quality of Life (Zeist, the Netherlands). The local medical ethics committee approved the protocol, and all volunteers gave their written informed consent. Volunteers were recruited from September to October 2001. Eligible volunteers were healthy as assessed by a general health and lifestyle questionnaire, blood pressure measurement, and blood analyses of haematology, homocysteine, B vitamins, liver enzymes, creatinine, glucose, and lipids. Plasma total homocysteine concentrations were below 25 μmol/l. Volunteers had no history of CVD, and had not used vitamin B supplements more than once a week in the 3 mo before entering the study. Out of the eligible participants, the 76 participants (44 males and 32 females, age 30–50 y) with the highest plasma total homocysteine concentrations (range 8.4 to 22.2 μmol/l) were included in this placebo-controlled, double-blind parallel trial (Figure 3). Volunteers were stratified by gender, plasma homocysteine concentration, blood pressure, and smoking (yes or no), and then randomly assigned to one of four treatment groups for 6 wk: (a) 1.5 g/d of anhydrous betaine (BUFA) (*n* = 19), (b) 3 g/d of betaine (*n* = 19), (c) 6 g/d of betaine (*n* = 19), or (d) placebo (*n* = 19). One person at the local pharmacy assigned codes to the study treatments and provided the key in a sealed envelope to the principal investigator at TNO. The statistician then randomly allocated treatment codes to the participant numbers. Randomization was done using a computerized procedure that produced combinations based on random seed numbers. The statistician supplied the principal investigator with sealed envelopes with the treatment allocation per participant. The participants and all others involved in this study were unaware of treatment allocation. The statistician performed unblinding of the treatment allocation after the study had ended, laboratory analyses were complete, and datasets were locked. The study supplements were dissolved in water and ingested on two daily occasions, one half of the daily dose after breakfast and the other half after the evening meal. Throughout the study participants were asked not to consume liver products more than twice a week, and not to consume more than two eggs per week. Six grams per day of betaine is the lowest therapeutic dose used to lower plasma homocysteine in hyperhomocysteinemic patients with genetic defects in their homocysteine metabolism [20]. We chose to study doses of betaine lower than 6 g/d, e.g., 1.5 g/d and 3 g/d, because these are within the range of intake of betaine with foods. Intake of betaine with foods is generally estimated at 0.5–2 g/d.

#### Blood sampling and blood lipid analysis

Venous blood was taken from the anticubital vein in the forearm following an overnight fast at baseline and after 2 wk and 6 wk of intervention. Samples were mixed and put on ice immediately after collection. Within 30 min samples were centrifuged for 10 min at 2,000 *g* at 4 °C. Samples were stored below −18 °C. Samples were coded to hide the identity and treatment of participants. All samples obtained from one participant were analyzed in the same run. We measured blood lipid concentrations in fasting EDTA plasma samples at baseline and after 2 wk of intervention, and in fasting heparin plasma samples collected at baseline and after 6 wk of intervention. Concentrations of total cholesterol, HDL cholesterol, and triacylglycerol were measured with a Hitachi 911 analyzer and enzymatic assays of Roche. LDL cholesterol concentrations were calculated with the formula of Friedewald et al. [19].

### Study 3

#### Participants and design

**Figure 4:**
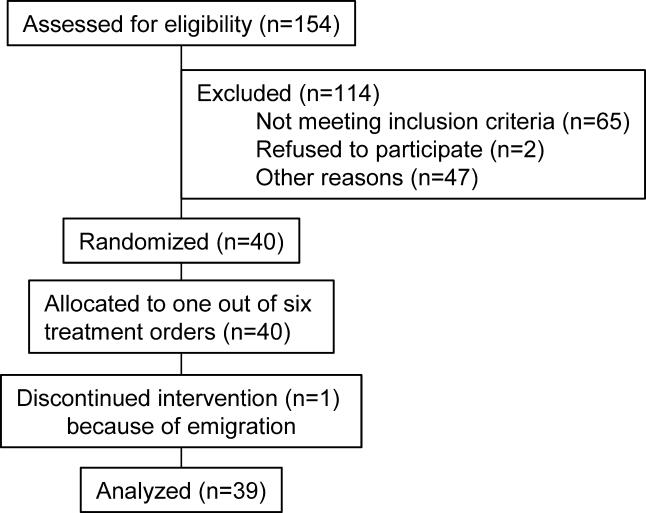
Flow Diagram of Participant Progress through Study 3

The primary endpoints of this study were plasma homocysteine concentrations and vascular function. Blood lipid measurements were planned before the study took place, but power analysis was based on changes in the primary endpoint vascular function, and not on changes in blood lipids. The original study protocol of this study can be found in Protocol S1, and the trial was registered at clinicaltrials.gov under identifier NCT00102843. The study was conducted at the division of Human Nutrition, Wageningen University (Wageningen, the Netherlands). The local medical ethics committee approved the protocol, and all volunteers gave their written informed consent. Volunteers were recruited from June to September 2002. Eligible volunteers were healthy as assessed by routine medical screening and a general health questionnaire, had plasma total homocysteine concentrations below 26 μmol/l, had no history of CVD; and had not used vitamin B supplements more than once a week in the 3 mo before entering the study. Out of the eligible participants, the 40 participants (23 males and 17 females, age 50–70 y) with the highest plasma total homocysteine concentrations (range 10.2 to 21.7 μmol/l) were included in this placebo-controlled, double-blind cross-over trial (Figure 4). Participants were randomly assigned to one out of six treatment orders, and they received each of the following supplements for 6 wk, with a 6-wk wash out in between: (a) 6 g/d of betaine (BUFA), (b) 0.8 mg/d of folic acid, or (c) placebo. A person not further involved in the study assigned codes to the study treatments, and randomly allocated the selected participants to one out of six treatment orders, according to a computer-generated randomization list, and kept the key in a sealed envelope. The participants and all others involved in this study were unaware of treatment allocation. The principal investigator performed unblinding of the treatment allocation after the study had ended and laboratory analyses were complete. The study supplements were dissolved in water and ingested twice per day, one half of the daily dose after breakfast and the other half after the evening meal. During the study participants were not allowed to consume supplements containing B vitamins, antioxidant vitamins (A, beta-carotene, C, and E), or omega-3 fatty acids/fish oil supplements.

#### Blood sampling and laboratory analyses

Venous blood was taken from the antecubital vein following an overnight fast on days 41 and 43 of each treatment period. Blood was collected in vacutainer tubes containing clot activator and a gel to separate serum and cells. About 30 min after collection, samples were centrifuged for 15 min at 2,000 *g* at 4 °C. All serum samples were stored below −70 °C. Samples were coded to hide the identity and treatment of participants. All samples obtained from one participant were analyzed in the same run. In serum samples we measured concentrations of total cholesterol, HDL cholesterol, and triacylglycerols on a Synchron LX 20 system (Beckman Coulter, Mijdrecht, the Netherlands). LDL cholesterol concentrations were calculated with the formula of Friedewald et al. [19].

### Study 4

#### Participants and design

**Figure 5:**
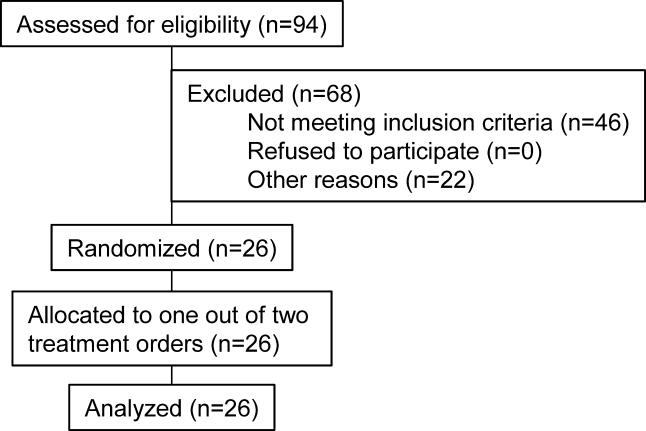
Flow Diagram of Participant Progress through Study 4

The primary endpoint of study 4 was plasma homocysteine concentrations. Blood lipid measurements were planned before the study took place, but power analysis was based on changes in the primary endpoint homocysteine concentrations, and not on changes in blood lipids. The original study protocol of this study can be found in Protocols S2–S4, and the trial was registered at clinicaltrials.gov under identifier NCT00102232. This study was conducted according to Good Clinical Practice guidelines at TNO Quality of Life (Zeist, the Netherlands). The local medical ethics committee approved the protocol, and all volunteers gave their written informed consent. Volunteers were recruited from March to May 2003. Eligible volunteers were healthy as assessed by physical examination, a general health and lifestyle questionnaire, blood pressure measurement, routine clinical laboratory tests, and blood analyses of homocysteine and B vitamins. Plasma homocysteine concentrations were below 26 μmol/l. Volunteers had no history of CVD, and had not used vitamin B supplements, lecithin, or supplements containing choline, choline derivatives, or betaine more than once a week during the 1 mo before screening. Out of the eligible men, the 26 men between 50–71 y of age with the highest plasma homocysteine concentrations (range 11.0 to 23.1 μmol/l) were included in this placebo-controlled, double-blind cross-over trial (Figure 5). Participants were stratified by plasma homocysteine concentrations at screening and by smoking habits and then randomly assigned to one of two treatment orders; they received each of the following supplements for 2 wk, with a 2-wk wash out in between: (a) 34.0 g of a soybean lecithin preparation, in which phosphatidylcholine is the only phospholipid (PhosChol Nutrasal, Oxford, Connecticut, United States) and (b) placebo oil, which consisted of 25.5 g of a mixture of edible oils that mimicked the fatty acid composition of the phosphatidylcholine supplement (provided by Unilever Research Laboratory, Vlaardingen, the Netherlands) (Table 2). A person not further involved in the study assigned codes to the study treatments and provided the key in a sealed envelope to the principal investigator at TNO. The statistician randomly allocated the selected participants to one of the two treatment orders. Randomization was done using a computerized procedure that produced combinations based on random seed numbers. The statistician supplied the principal investigator with sealed envelopes with the treatment allocation per participant. The participants and all others involved in this study were unaware of treatment allocation. The statistician performed unblinding of the treatment allocation after the study had ended, laboratory analyses were complete, and datasets were locked. Phosphatidylcholine and placebo supplements were matched for fat content and fatty acid composition (Table 2). The amount of choline in 34 g of the phosphatidylcholine supplement was 2.6 g, as measured by Koc et al. [21] and by TNO. The daily dose of 2.6 g of choline is well below the current tolerable upper intake level of 3.5 g of choline per day for adults [22]. Participants ingested half of the daily supplement dose two times per day (i.e., at breakfast and at dinner). The individual portions of half the daily dose of the supplements were mixed with 200 ml of custard. Participants returned 99.5% of the bowls empty, which indicated good compliance. From 2 wk before the start of the study until the end of the study participants were not allowed to consume food products rich in lecithin or betaine.

**Table 2:**
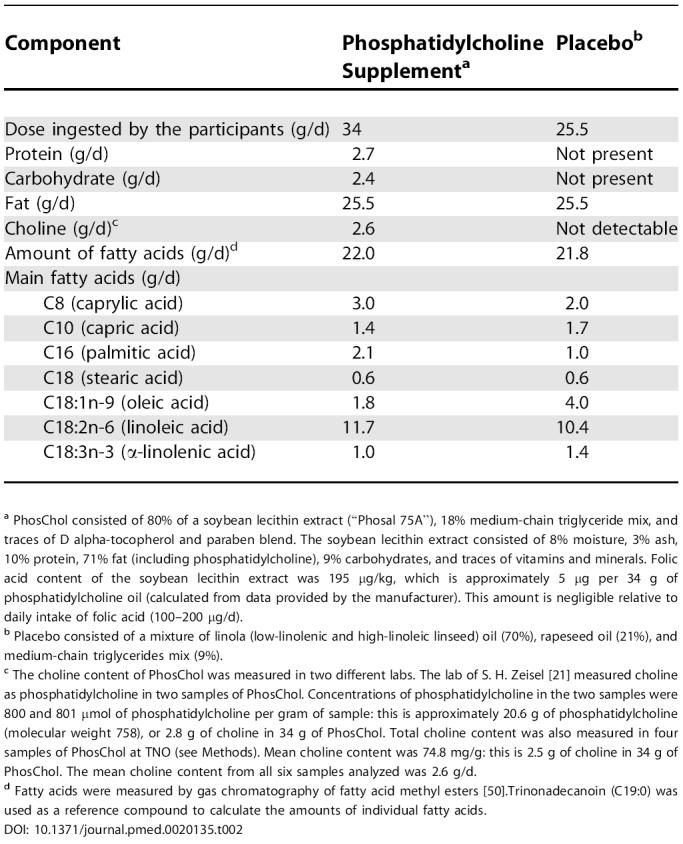
Composition of Treatment Supplements in Study 4

^a^ PhosChol consisted of 80% of a soybean lecithin extract (“Phosal 75A”), 18% medium-chain triglyceride mix, and traces of D alpha-tocopherol and paraben blend. The soybean lecithin extract consisted of 8% moisture, 3% ash, 10% protein, 71% fat (including phosphatidylcholine), 9% carbohydrates, and traces of vitamins and minerals. Folic acid content of the soybean lecithin extract was 195 μg/kg, which is approximately 5 μg per 34 g of phosphatidylcholine oil (calculated from data provided by the manufacturer). This amount is negligible relative to daily intake of folic acid (100–200 μg/d).

^b^ Placebo consisted of a mixture of linola (low-linolenic and high-linoleic linseed) oil (70%), rapeseed oil (21%), and medium-chain triglycerides mix (9%).

^c^ The choline content of PhosChol was measured in two different labs. The lab of S. H. Zeisel [21] measured choline as phosphatidylcholine in two samples of PhosChol. Concentrations of phosphatidylcholine in the two samples were 800 and 801 μmol of phosphatidylcholine per gram of sample: this is approximately 20.6 g of phosphatidylcholine (molecular weight 758), or 2.8 g of choline in 34 g of PhosChol. Total choline content was also measured in four samples of PhosChol at TNO (see Methods). Mean choline content was 74.8 mg/g: this is 2.5 g of choline in 34 g of PhosChol. The mean choline content from all six samples analyzed was 2.6 g/d.

^d^ Fatty acids were measured by gas chromatography of fatty acid methyl esters [50].Trinonadecanoin (C19:0) was used as a reference compound to calculate the amounts of individual fatty acids.

#### Blood sampling and laboratory analyses

Venous blood was taken from the antecubital vein following an overnight fast on days 13 and 15 of each treatment period. Blood was collected in vacutainer tubes containing clot activator and a gel to separate serum and cells. About 30 min after collection, samples were centrifuged for 15 min at 2,000 *g* at 4 °C. All serum samples were stored below −70 °C. Samples were coded to hide the identity and treatment of participants. All samples obtained from one participant were analyzed in the same run. In serum samples we measured concentrations of total cholesterol, HDL cholesterol, and triacylglycerols with a Hitachi 911 analyzer and enzymatic assays of Roche. LDL cholesterol concentrations were calculated with the formula of Friedewald et al. [19]. For the measurement of choline at TNO, choline was liberated from the sample by boiling the sample for 4 h with nitric acid (17 v/v%). After filtration of the sample, the pH of the extract was raised to 9.0 with sodium hydroxide solution. To an aliquot part of the extract a solution of ammonium reineckate in methanol was added, which formed the insoluble choline reineckate. The precipitate was filtered off and dissolved in acetone. The color of the acetone solution was determined with a spectrophotometer at a wavelength of 530 nm.

### Statistics

The sample size of each study was based on power analysis for the primary outcome measure. To exclude effects of the matrix in which lipids were measured [23], all changes in lipid concentrations within persons or studies were based on measurements within the same matrix. So choice of matrix could not have been responsible for differences in results between the studies. First we calculated the mean change in lipid concentrations relative to placebo after supplementation with the treatments for each study. Means were compared with the General Linear Models procedure in SAS (ANOVA; SAS version 6.12, SAS Institute, Cary, North Carolina, United States). For the parallel studies 1 and 2, Dunnett's two-tailed *t*-test was used to test whether any of the treatments significantly differed from placebo. For the cross-over studies 3 and 4, a paired Student's *t*-test was used to test whether any of the treatments significantly differed from placebo. Then we combined the lipid data of the studies with betaine or folic acid supplementation. For this we calculated a combined effect of all four studies, which was a weighted mean and 95% confidence intervals of the change in lipid concentrations relative to placebo after betaine supplementation (studies 1, 2, and 3), and after folic acid supplementation (studies 1 and 3) according to the method used by Curtin et al. [24] (Tables 3 and 4). We tested whether the weighted mean changes differed from zero with a *t*-test. For all tests mean differences were considered statistically significant at *p* < 0.05.

**Table 3:**
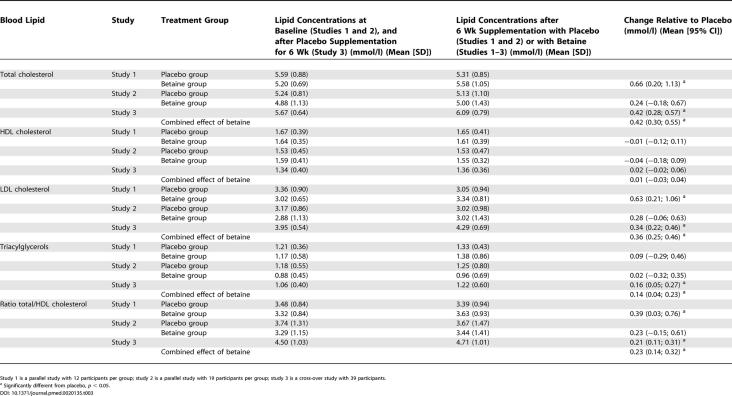
Mean Changes in Blood Lipid Concentrations and the Ratio of Total to HDL Cholesterol in Fasting State in Studies 1, 2, and 3, and the Combined Effect of Supplementation with 6 g/d of Betaine

Study 1 is a parallel study with 12 participants per group; study 2 is a parallel study with 19 participants per group; study 3 is a cross-over study with 39 participants.

^a^ Significantly different from placebo, *p* < 0.05.

**Table 4:**
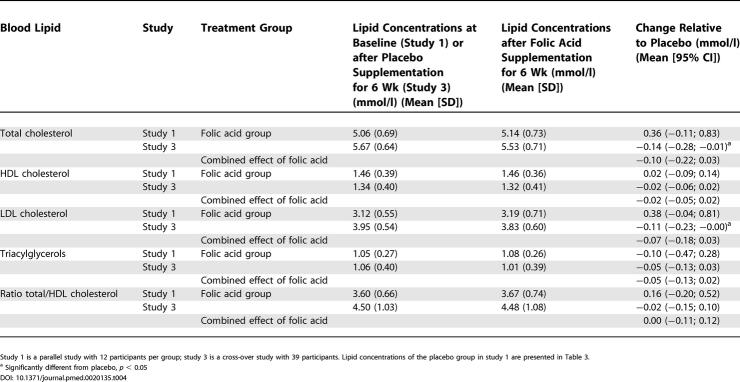
Mean Changes in Blood Lipid Concentrations and the Ratio of Total to HDL Cholesterol in Fasting State in Studies 1 and 3, and the Combined Effect of Supplementation with 0.8 mg/d of Folic Acid

Study 1 is a parallel study with 12 participants per group; study 3 is a cross-over study with 39 participants. Lipid concentrations of the placebo group in study 1 are presented in Table 3.

^a^ Significantly different from placebo, *p* < 0.05

## Results

All participants completed the study, except for one male participant who withdrew from study 3 due to emigration. No serious adverse events were reported in any of the studies. Non-serious adverse events are shown in Table S5.

**Figure 6:**
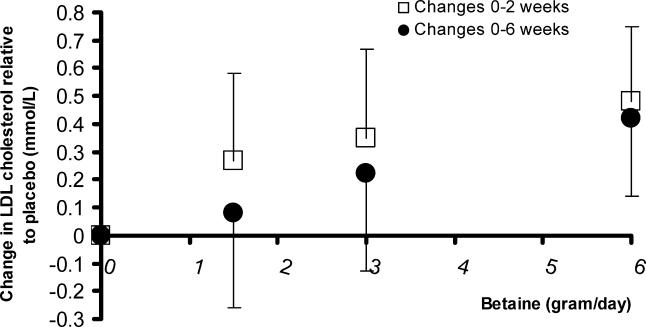
Mean Change Relative to Placebo in LDL Cholesterol Concentrations Compared are the values after participants had ingested 1.5 g/d of betaine (study 2, *n =* 19), 3 g/d of betaine (study 2, *n =* 18), or 6 g/d of betaine (studies 1 and 2 combined, *n =* 31) after 2 wk (+95% confidence interval) and 6 wk (–95% confidence interval). In the group that ingested 3 g/d of betaine one participant missed the blood collection before treatment.

Supplementation of 6 g/d of betaine for 6 wk increased total cholesterol concentrations by 0.42 mmol/l (8%) relative to placebo (see Table 3). This increase was almost completely accounted for by an increase in LDL cholesterol concentrations of 0.36 mmol/l (11%). Betaine increased triacylglycerol concentrations by 0.14 mmol/l (13%). HDL cholesterol concentrations did not change, but the ratio of total to HDL cholesterol concentration increased by 0.23 (6%). The increases in LDL cholesterol concentrations in the groups that ingested 1.5 g/d of betaine and 3 g/d of betaine for 6 wk were higher than in the placebo group, but these changes did not reach statistical significance (Figure 6). The effects of betaine supplementation on LDL cholesterol concentrations were already evident after 2 wk of supplementation in studies 1 and 2.

Supplementation of 0.8 mg/d of folic acid for 6 wk did not affect lipid concentrations in healthy volunteers (see Table 4).

Supplementation with 2.6 g/d of choline (provided as phosphatidylcholine) for 2 wk increased serum triacylglycerols by 0.14 mmol/l (8%), but did not affect cholesterol concentrations (Table 5).

**Table 5:**
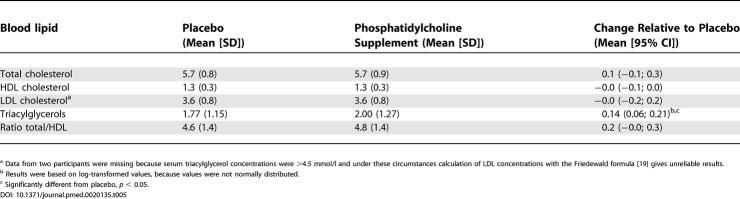
Concentrations of Serum Lipids (mmol/l) and the Ratio of Total to HDL Cholesterol in Fasting State after 26 Healthy Men Had Ingested Phosphatidylcholine Providing ~2.6 g/d of Choline or Placebo for 2 Wk

^a^ Data from two participants were missing because serum triacylglycerol concentrations were >4.5 mmol/l and under these circumstances calculation of LDL concentrations with the Friedewald formula [19] gives unreliable results.

^b^ Results were based on log-transformed values, because values were not normally distributed.

^c^ Significantly different from placebo, *p* < 0.05.

## Discussion

We found that supplementation of 6 g/d of betaine for 6 wk consistently increased total cholesterol concentrations, mainly LDL cholesterol, and triacylglycerol concentrations. Phosphatidylcholine supplementation for 2 wk increased only serum triacylglycerol concentrations. Folic acid supplementation did not affect lipid concentrations.

The three studies on betaine supplementation were not originally designed to investigate changes in blood lipids. Therefore, we did not perform power calculations for changes in blood lipids for each study beforehand. We combined the lipid data of the three studies to have a better estimate of the effects of betaine on blood lipids, and thereby increase the statistical power afterwards. Similarly we did not do power calculations for effects of folic acid on blood lipids. The increase in LDL cholesterol concentrations with betaine supplementation that we found in healthy humans is in accordance with results from previous studies in renal patients [13] and obese individuals who were on a weight-loss diet [9]. Doses of betaine lower than 6 g/d also raised LDL cholesterol, although this did not reach statistical significance (Figure 6). Our study was not designed to investigate the dose–response relationship between betaine doses and changes in LDL cholesterol. Establishing the dose–response relationship between betaine and LDL cholesterol might require more than three betaine dose groups*.* Furthermore, betaine supplementation affected blood lipids already after 2 wk of intervention (Figure 6). The increase in triacylglycerol concentrations with betaine supplementation is well in line with the results of Schwab et al. in obese individuals [9]. In the obese individuals betaine supplementation increased triacylglycerol concentrations by approximately 12%, although this increase did not reach statistical significance.

Our observations should be of specific concern for clinical practice as well as for the general population. Patients with inborn errors in the enzymes involved in homocysteine metabolism and who are not or only partially responsive to pyridoxine are generally prescribed betaine in doses of 6 g/d or higher in order to lower their plasma homocysteine [25]. Our data imply that betaine treatment in these patients may have adverse effects on lipid concentrations. No one has systematically reported changes in serum cholesterol concentrations in hyperhomocysteinemic patients who are on betaine treatment, although this may be warranted. Clearly in these patients, the benefit of the substantial homocysteine-lowering effect of betaine outweighs the effect of any modest lipid-related changes. Furthermore, our data imply that among the general population intake of extra betaine, e.g., from supplements or through increased intake of betaine-rich foods, may not bring the expected benefits for CVD prevention predicted from the homocysteine-lowering effect of betaine.

Phosphatidylcholine supplementation increased serum triacylglycerols, but did not affect serum cholesterol concentrations. We supplemented phosphatidylcholine for only 2 wk, and it is possible that this was too short to induce changes in serum cholesterol, although betaine supplementation did increase blood cholesterol concentrations within 2 wk. We carefully matched the fat content and fatty acid composition of the control and the phosphatidylcholine supplements. Previous studies that also attempted to control for the fatty acid component of lecithin (the trivial name for phosphatidylcholine) [26–30], or that tested choline bitartrate [31], suggest that there is little or no effect of the choline moiety of lecithin on blood lipids, provided that the fatty acids in lecithin are balanced in the placebo treatment. Only two of the studies that investigated phosphatidylcholine included a concurrent control group [27,30]. In the randomized cross-over study of Childs et al. [27] triacylglycerol concentrations in 12 normolipidemic individuals were 0.01 mmol/l lower after they had consumed lecithin for 3 wk than after they had consumed placebo. Oosthuizen et al. [30] found in a parallel study with seven hyperlipidemic men per group that the median triacylglycerol concentration decreased by 0.19 mmol/l in the lecithin group relative to the placebo group after 2 wk of intervention. After 4 wk of intervention this difference had disappeared. The increase in triacylglycerol concentrations with phosphatidylcholine treatment in our study could be a chance finding. However, our study was larger than previous studies, we used a cross-over design with a wash out period, and participants received supplements in random order. Moreover, the dose of phosphatidylcholine we used was higher than in the previous studies. Therefore, our study leaves open a real possibility that the choline moiety of phosphatidylcholine in high doses raises serum triacylglycerol concentrations.

We propose that the mechanism by which betaine and phosphatidylcholine supplementation increase blood lipid concentrations involves increased export of lipids from the liver into the circulation. Lipids are exported from the liver through VLDL particles, which consist of a triglyceride core packaged in an amphipatic shell of phospholipids, mostly phosphatidylcholine, and apolipoprotein B-100. More VLDL will increase transport of lipids from the liver into the blood, and concentrations of lipids in the liver decrease accordingly [11]. Betaine and phosphatidylcholine supplementation could increase VLDL synthesis via increased availability of phosphatidylcholine, an essential component of VLDL (see Figure 1) [32]. Furthermore, betaine might increase phosphatidylcholine synthesis owing to increased availability of S-adenosylmethionine via increased remethylation of homocysteine into methionine, because S-adenosylmethionine is the methyl donor for the formation of phosphatidylcholine out of phosphatidylethanolamine [33]. In addition, betaine supplementation could promote phosphatidylcholine synthesis through increasing the activity of the enzyme betaine–homocysteine methyltransferase [34]. Betaine–homocysteine methyltransferase catalyzes the transfer of a methyl group from betaine to homocysteine, but it also catalyzes the first step in the three-enzyme pathway that promotes methylation of phosphatidylethanolamine into phosphatidylcholine [35,36].

We speculate that betaine and phosphatidylcholine supplementation increase lipid concentrations in blood because of increased synthesis and availability of phosphatidylcholine, which promotes VLDL secretion from the liver into plasma, resulting in higher lipid concentrations in blood [32,37].

Our data indicate that folic acid supplementation does not affect serum lipid concentrations, which is in agreement with other studies [38–41].

The consequences of betaine or phosphatidylcholine supplementation for risk of CVD are difficult to predict. In healthy participants, betaine supplementation lowers plasma homocysteine after methionine loading by 20%–40%. Betaine as well as phosphatidylcholine supplementation lower fasting plasma homocysteine by 12%–20% (Table 6) [7,8]. This is estimated to lower CVD risk by 10%–15% [1,42]. However, it is not yet firmly established that homocysteine lowering indeed lowers risk of CVD, although evidence for a causal relationship is accumulating [1–3,43]. We now find that betaine not only decreases homocysteine, but also increases LDL cholesterol by approximately 10% and the total/HDL cholesterol ratio by approximately 6%. This is estimated to increase CVD risk by approximately 10% [44–46]. Betaine (6 g/d) and phosphatidylcholine (providing approximately 2.6 g/d of choline) supplementation increase triacylglycerol concentrations by up to 13%, which is estimated to increase risk of CVD by approximately 6%, after adjustment for other risk factors [47–49]. This implies that betaine supplementation is expected to increase risk of CVD if homocysteine is not a cause of CVD, or does not affect risk if homocysteine is causally involved. Whether phosphatidylcholine affects CVD risk remains uncertain so far.

**Table 6:**
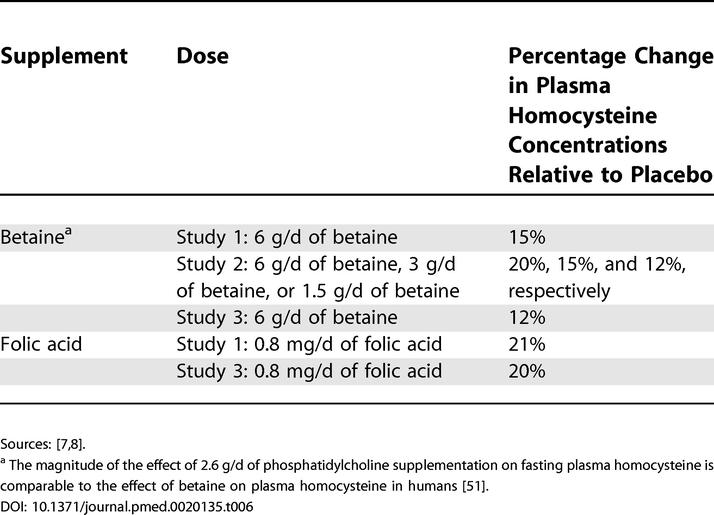
Percentage Change in Fasting Concentrations of Plasma Homocysteine after Participants Had Ingested Betaine and Folic Acid for 6 Wk

Sources: [7,8].

^a^ The magnitude of the effect of 2.6 g/d of phosphatidylcholine supplementation on fasting plasma homocysteine is comparable to the effect of betaine on plasma homocysteine in humans [51].

Observational studies could help in assessing the overall effect of phosphatidylcholine and betaine consumption on CVD risk. Recently, data on betaine and choline contents in foods became available [10], so it should now be feasible to assess the relationship between betaine and phosphatidylcholine intake and risk of CVD. In summary, the adverse effects of betaine on blood lipids make it less suitable as a homocysteine-lowering agent in healthy humans. Phosphatidylcholine may be more suitable because it only slightly increases serum triacylglycerol concentrations and does not affect LDL cholesterol. Folic acid has no adverse side effects on blood lipids, and therefore folic acid supplementation should remain the preferred homocysteine-lowering treatment in healthy humans.

## Supporting Information

Protocol S1 Protocol for Study 3 (95 KB DOC).

Protocol S2 Protocol for Study 4 (840 KB PDF).

Protocol S3 Amendment 1 of Protocol for Study 4 (585 KB PDF).

Protocol S4 Amendment 2 of Protocol for Study 4 (470 KB PDF).

Table S1 Consort Checklist for Study 1 (47 KB DOC).

Table S2 Consort Checklist for Study 2 (47 KB DOC).

Table S3 Consort Checklist for Study 3 (47 KB DOC).

Table S4 Consort Checklist for Study 4 (38 KB DOC).

Table S5 Overview of Non-Serious Adverse Events per Study (25 KB DOC).

### Trial Registration

Study 3 is registered at clinicaltrials.gov under identifier NCT00102843. Study 4 is registered at clinicaltrials.gov under identifier NCT00102232.

**Patient Summary**

**Background**

Homocysteine is an amino acid in the blood (amino acids are the building blocks of proteins). Too much homocysteine in the blood is related to a higher risk of stroke and heart disease. Mildly elevated levels (which are quite common) might promote atherosclerosis (furring up of the arteries), but this link has not been proven yet. Studies are currently under way to test whether reducing homocysteine levels in people who have mildly elevated levels could prevent heart disease or stroke. Again, the results are not yet known.

**Why Was This Study Done?**

In people who have dramatically elevated levels of homocysteine, different food supplements have been shown to bring down homocysteine concentrations and to reduce the risk of heart disease and strokes. These supplements include folic acid, vitamin B12, vitamin B6, betaine, and phosphatidylcholine. This study looked at the effects of betaine, folic acid, and phosphatidylcholine on blood lipids in healthy people. The researchers wanted to study blood lipids, especially “bad” cholesterol (LDL cholesterol), because they are known to influence the risk of heart disease.

**What Did the Researchers Do?**

They analyzed data from four different studies that looked at the ability of the three substances to lower homocysteine levels. They analyzed the blood samples (which had previously been used to measure homocysteine) for lipid levels.

**What Did They Find?**

They found that betaine increased the level of “bad” cholesterol. Folic acid did not affect lipid levels. The data for phosphatidylcholine were not conclusive.

**What Does This Mean?**

This suggests that the beneficial effects of betaine (which lowers homocysteine) might be undone at least in part by its negative effects on blood lipids. Based on these results, folic acid would be a better choice for people who want to lower their homocysteine levels, since folic acid doesn't cause a rise in “bad” cholesterol.

**More Information Online**

More information on the link between homocysteine and heart disease can be found at the following Web sites. Web site of the American Heart Association (search for “homocysteine” or “cholesterol”): http://www.americanheart.org/presenter.jhtml?identifier = 1200000

Web site of the Australian Heart Association (search for “homocysteine,” “cholesterol,” and “cardiovascular risk”): http://www.heartfoundation.com.au/

Web site of the British Heart Foundation (search for “homocysteine,” “cholesterol,” and “cardiovascular risk”): http://www.bhf.org.uk/

WebMD Web pages on homocysteine, cholesterol, and heart disease (or search http://webmd.com for “homocysteine” or “cholesterol”): http://my.webmd.com/content/pages/9/1675_57859; http://my.webmd.com/content/pages/9/1675_57815

## Abbreviations

CVD: cardiovascular disease
HDL: high-density lipoprotein
LDL: low-density lipoprotein
VLDL: very low density lipoprotein
