# Supplementary material for: Effect of Homocysteine-Lowering Nutrients on Blood Lipids: Results from Four Randomised, Placebo-Controlled Studies in Healthy Humans
Source: PLoS Med. 2005 May 31;2(5):e135. doi: 10.1371/journal.pmed.0020135 (PMC1140947; doi:10.1371/journal.pmed.0020135)
Supplement: Protocol S1 — (95 KB DOC). [file pmed.0020135.sd001.doc]

# **Protocol**

Effect of lowering of fasting plasma homocysteine concentrations on endothelial function in healthy volunteers

Causality of homocysteine in cardiovascular disease

Wageningen Centre for Food Sciences (WCFS)

P.O. Box 8129

6700 EV, Wageningen

The Netherlands

Index

Preface [3](#__RefHeading___Toc6131941)

Introduction [3](#__RefHeading___Toc6131942)

Objective [5](#__RefHeading___Toc6131943)

Study design [5](#__RefHeading___Toc6131944)

Subjects [5](#__RefHeading___Toc6131945)

Inclusion criteria [5](#__RefHeading___Toc6131946)

Exclusion criteria [6](#__RefHeading___Toc6131947)

Supplements [7](#__RefHeading___Toc6131948)

Blinding [7](#__RefHeading___Toc6131949)

Safety of the supplements [7](#__RefHeading___Toc6131950)

Restrictions during the study [7](#__RefHeading___Toc6131951)

Selection of volunteers [8](#__RefHeading___Toc6131952)

Measurements [9](#__RefHeading___Toc6131953)

Pre-study measurements: screening [9](#__RefHeading___Toc6131954)

In-study measurements [9](#__RefHeading___Toc6131955)

Power calculation [10](#__RefHeading___Toc6131956)

FMD [10](#__RefHeading___Toc6131957)

Plasma homocysteine [10](#__RefHeading___Toc6131958)

Compensation for volunteers [10](#__RefHeading___Toc6131959)

Advantage for participants of the study [10](#__RefHeading___Toc6131960)

Insurance [11](#__RefHeading___Toc6131961)

Submission personal data to CBP (Dutch Data Protection authority) [11](#__RefHeading___Toc6131962)

Appendix I - Flow mediated vasodilation (FMD) measurement [12](#__RefHeading___Toc6131963)

Appendix II - Blood pressure measurement [13](#__RefHeading___Toc6131964)

References [14](#__RefHeading___Toc6131965)

# Preface

The current study will be performed at the division of Human Nutrition and Epidemiology, Wageningen University, within the programme Nutrition and Health of WCFS (Wageningen Centre for Food Sciences).

The current study is one of two studies within a larger WCFS project on homocysteine and endothelial function. Both studies aim at determining the causality of homocysteine in cardiovascular disease:

1. In study A (current study) we will determine whether lowering of fasting plasma homocysteine by two independent metabolic pathways improves endothelial function.
2. In study B we will determine whether reducing the increase in plasma homocysteine after an oral methionine load by three independent metabolic pathways improves endothelial function.

A high fasting plasma homocysteine concentration and a high increase in homocysteine after a methionine load are both associated with cardiovascular disease. Endothelial function is recognized as an early risk marker of cardiovascular disease. It can be measured via Flow Mediated vasoDilation (FMD), a noninvasive measurement with high resolution ultrasound.

The current protocol concerns Study A. Study B is described in a separate protocol.

# Introduction

Cardiovascular disease is the most important cause of death in the western world. A high fasting plasma homocysteine is a potential risk factor for cardiovascular disease and death: an increase in fasting plasma homocysteine of 5 μmol/L increases the risk of cardiovascular disease by 20-30% (1). Due to the observational character of the epidemiologic studies, reverse causality cannot be excluded. Therefore, it remains unsure whether homocysteine is a causal factor for cardiovascular disease. Homocysteine-lowering with B-vitamin supplementation reduces restenosis in the coronary arteries of patients who underwent angioplasty (2), which supports the hypothesis that homocysteine is causal for recurrent cardiovascular disease. Other long-term homocysteine-lowering intervention studies in cardiovascular disease patients are ongoing (3, 4).

Studies in apparently healthy individuals on the effects of homocysteine-lowering on cardiovascular disease risk are few. In our research group we currently investigate the effect of homocysteine-lowering through folic acid supplementation on carotid artery intima media thickness (IMT), a marker of atherosclerosis (J. Durga, P. Verhoef et al.).

The mechanism underlying the effect of homocysteine on the cardiovascular system is still unknown. High homocysteine concentrations might cause endothelial dysfunction. Endothelial dysfunction indicates an early stage of disease and can be measured non-invasively in humans through Flow Mediated vasoDilation in the brachial artery (FMD). FMD is the increase in arterial diameter that is induced by a local increase in arterial blood flow. The expansion of the artery upon increased blood flow is measured with high resolution ultrasound (5). FMD is a promising biomarker that indicates risk of cardiovascular disease (6-8). In the current study we will use FMD as a biomarker for cardiovascular disease risk in healthy volunteers. Previous studies showed that FMD responds rapidly to dietary interventions (9-11).

A high concentration of homocysteine in blood is related to an impaired endothelial function. This was shown in animal studies with diet-induced hyperhomocysteinemia, (12) in hyperhomocysteinemic subjects (13-16), in healthy humans with hyperhomocysteinemia after a methionine loading (17-19), and after ingestion of homocysteine (20).

Lowering of plasma homocysteine through folic acid supplementation improves FMD in most studies (21-27), but not all (28-30). However, there are indications that folic acid improves endothelial function via mechanisms independent from homocysteine (19, 26, 27, 31).

The objective of this study is to investigate whether lowering of fasting homocysteine concentrations by two independent metabolic pathways improves endothelial function in healthy volunteers.

To achieve this, plasma homocysteine will be lowered via two independent metabolic pathways. The rationale is that if homocysteine-lowering via two independent pathways both improve risk for cardiovascular disease then homocysteine is likely to be the causal factor, i.e. independent effects of the treatment itself become unlikely.

In the current study folic acid and betaine will be used as homocysteine-lowering agents. Folic acid and betaine both lower plasma homocysteine via independent remethylation pathways (Figure). The methyl group necessary for remethylation of homocysteine into methionine can be derived from the folic acid metabolite, 5-methyltetrahydrofolate, or from betaine (figure). Our group previously showed that supplementation of betaine and folic acid for 6 weeks lowers fasting plasma homocysteine concentrations by 15-20% (32).

Figure: Homocysteine metabolism

# Objective

Investigate whether lowering of fasting homocysteine concentrations by two independent metabolic pathways improves endothelial function in healthy volunteers.

The two homocysteine-lowering treatments are folic acid and betaine. The rationale is that if homocysteine-lowering via two independent pathways both improve risk for cardiovascular disease then homocysteine is likely to be the causal factor, i.e. independent effects of the treatment itself become unlikely.

# Study design

- Randomized, blind, cross over study
- Intervention period 6 weeks; wash out period 6 weeks
- Main outcome: endothelial function after an overnight fast at the end of each 6-weeks supplement period. Endothelial function is measured as flow mediated vasodilation (FMD). FMD is the increase in arterial diameter that is induced by a local increase in arterial blood flow, measured with high resolution ultrasound

|  | **Week** | | | | | |
| --- | --- | --- | --- | --- | --- | --- |
|  | **−6** | **0-6** | **7-12** | **13-18** | **19-24** | **25-30** |
|  |  |  |  |  |  |  |
| Supplement intake* |  | Betaine | Wash out  No supplement | Folic acid | Wash out  No supplement | Placebo |
| Measurements  Pre-study (screening) | 🡹 |  |  |  |  |  |
| Measurements in-study |  | 🡹🡹 |  | 🡹🡹 |  | 🡹🡹 |

* Example of treatment order is given, but supplements will be ingested by each volunteer in random order

# Subjects

Forty healthy men and women 50-70 years with a relatively high concentration of plasma homocysteine will participate. We aim at enrolling approximately 80 eligible men and women for the screening measurement and then select 40 subjects with the highest homocysteine concentrations (at least 40% of each gender).

## Inclusion criteria

- Apparently healthy
- Age: 50-70 y at day 1 of the study
- women postmenopausal: ≥ 2 y after last menstruation. If the uterus was surgically removed, the women must be ≥ 55 y
- Normal blood values for:
  - hematology
  - Total homocysteine
  - lipids: total cholesterol and triglycerides
  - vitamin B6
  - vitamin B12
  - Folic acid
  - Liver enzymes (aminotransferase concentrations) (assessment liver function)
  - creatinine (assessment renal function)
    NB. Cut off values depend on the laboratory where samples are measured
- Absence of protein and glucose in urine sample
- 18 kg/m^2^ ≤ BMI ≤ 30 kg/m^2^
- Good ultrasound visibility of the brachial artery, judged by the echografist.
- Willing not to use supplements containing B-vitamins, antioxidant vitamins (A, beta-carotene, C and E) or n-3 fatty acids/fish oil supplements from screening day (>2 months before start of the study) until end of study
- Willing not to be blood or plasmaferese donor from 4 weeks before the screening day, and 4 weeks before the start of the study until the end of study

## Exclusion criteria

- Any chronic or acute disease (e.g. diabetes, renal disease, inflammation)
- Current, or history of cardiovascular disease
- Hypertension
  Criteria for moderate hypertension WHO 1999:
  - Systolic > 160 mmHg or
  - Diastolic > 100 mmHg
- Medical history or surgical events know to interfere with the study
- Fasting plasma total homocysteine > 26 μmol/L
- Alcohol consumption:
  - women >21 consumptions /week
  - men >28 consumptions/week
- Weight loss or gain > 2 kg in the month prior to screening
- Any special diet (prescribed, slimming, macrobiotic or total vegetarian). Sole exclusion of meat and fish from a otherwise ‘normal’ western diet is allowed.
- Lactose intolerance
- Use of supplements containing B-vitamins more than once weekly in the period from 3 months before the screening day
- Participation in any other trial up to 3 months before this study
- Use of medication known to interfere with the study outcome e.g.:
  - Medication for hypertension
  - Hormone replacement therapy (women)
  - Medication interfering with homocysteine metabolism
  - Medication interfering with endothelial function
  - Medication interfering with digestion

# Supplements

The following supplements will be ingested daily for 6 weeks each in random order.

- folic acid: 800 μg/day + 5.9992 g placebo powder
- betaine: 6 g/day
- placebo (lactose): 6 g/day

Supplements will be ingested as a powder dissolved in water. The dose will be divided over 2 portions per day.

## Blinding

The study will be blinded as far as possible. In order to keep the study blind, every supplement will contain a total of 6 gram of powder. To achieve this, we will add placebo powder to the folic acid supplement up to 6 gram.

Furthermore, betaine has a specific taste whereas folic acid and placebo do not. Therefore, we will add a small amount of citric acid (sour) or quinine (bitter) to the folic acid supplement and placebo supplement in order to give all 3 supplements a different taste. This way, the volunteers as well as the researchers will be unable to identify the supplements.

## Safety of the supplements

Folic acid has been used in previous studies and is safe. The dose used in this study (800 μg/day of folic acid) is lower than the maximum dose of 1000 μg folic acid/day which is regarded as safe by the Dutch Health council.

Betaine occurs normally in the body and in foods and it is used as a flavor substance in the food industry, eg. in bread and pasta products. It is also a metabolite of choline metabolism. Choline also occurs normally in foods. The daily intake of betaine is 0.5-2 gram (personal communication with Prof. Steven Zeisel, Department of Nutrition, School of Public Health, University of North Carolina at Chapel Hill). The dose of 6 gram/day of betaine which we will use in the current study is also achievable through normal food intake. Further, this dose is used as homocysteine-lowering therapy in subjects with hyperhomocysteinemia and we also used this dose in our previous studies with healthy volunteers (32, 33)(Olthof et al., in preparation). In our previous studies only 1 person complained of a change in bowel habit, but it is unsure whether this is caused by the betaine supplement or not. No other adverse effects were reported during the previous studies with betaine. No other serious adverse effects of 6 gram/day of betaine are known.

# Restrictions during the study

#### Diet

During the study subjects are not allowed to consume supplements containing B-vitamins, antioxidant vitamins (A, beta-carotene, C and E) or n-3 fatty acids/fish oil supplements

Restrictions on the day before the measurement day

- On the day before each measurement day all subjects will receive a standard diet based on their normal energy intake. Breakfast, lunch, diner and snacks will be provided by the division of Human Nutrition and Epidemiology, Wageningen University. The diet will consist of normal products, but foods rich in protein, folic acid, betaine or choline will be avoided. Subjects are not allowed to consume any of their own foods during these days, except for coffee and tea which is allowed, but the amount should be constant over the days. Subjects can prepare and eat the foods at home.
- No coffee and tea is allowed after 18.00 h.
- Smoking is not allowed after 18.00 h
- The evening meal should be ingested before 20.00 h.
- after 22.00h subjects are not allowed to eat or drink, except water.

Restrictions on the measurement day

Subjects are not allowed to smoke, eat or drink (except water) until the FMD measurement has ended (in the morning). After the FMD measurement a breakfast without restrictions will be provided.

Other

Physical activity, dietary habit and smoking habit should remain constant during the study.

# Selection of volunteers

Subjects will be informed about the study orally (information meeting) and in writing (information brochure). Subjects will sign an informed consent before the start of the study.

Recruitment procedure:

1. Mailing of information to subjects from previous WCFS-studies that agreed to receive information about following studies (database with interested persons exist at WCFS) and to subjects recruited through local municipalities
   - Letter
   - Flyer with short description of the study
   - Application card for information
2. Mailing of information to subjects who returned the application card
   - Information brochure
   - Informed consent
   - Medical questionnaire
3. Invitation of subjects for information meeting, based on return of informed consent and eligibility criteria from medical questionnaire
4. Information meeting
5. Screening
   - height/weight measurement
   - blood sample
   - urine sample
6. Selection of subjects with highest total homocysteine concentrations will undergo
   - blood pressure measurement
   - image of brachial artery with ultrasound
7. Forty eligible subjects, based on in/exclusion criteria are included in the study

# Measurements

## Pre-study measurements: screening

- weight
- height
- blood pressure
- measurements in blood/serum/plasma:
- hematology
- total homocysteine
- vitamin B6
- vitamin B12
- folic acid
- lipids: total cholesterol and triglycerides
- liver enzymes
- creatinine
- urine: protein and glucose
- medical questionnaire

## In-study measurements

Measurements after an overnight fast:

- flow mediated dilatation (FMD), see appendix I
- blood pressure, according to a standardized protocol, see appendix II
- measurements in blood/serum/plasma:
- total homocysteine
- vitamin B6
- vitamin B12
- folate*
- betaine* (glycine betaine, C_5_H_11_NO_2_)
- Lipids**
- S-Adenosylmethionine (SAM) and S-adenosylhomocysteine (SAH)^#^
- Asymmetric dimethylarginine (ADMA)^$^
- weight

* Folate and betaine will be measured to check for compliance of supplement intake

** lipids (total cholesterol, LDL cholesterol, HDL cholesterol and triglycerides) will be measured to check whether they stay the same during the study. Changes in lipid concentrations might affect the FMD measurement.

^#^SAM and SAH are intermediates of the methylation pathway of methionine into homocysteine and are subject to change after folic acid and betaine supplementation. In addition SAH might also be an indicator of cardiovascular disease(34)

^$^ADMA is produced during the demethylation of methionine into homocysteine. ADMA might reduce Nitric oxide production because it is an inhibitor of the enzyme nitric oxide synthase. Nitric oxide is a potent vasodilator involved in endothelial function. ADMA might therefore be involved in the mechanism underlying the relation between homocysteine and endothelial function(35, 36).

# Power calculation

The number of volunteers in this study is based on the power calculation for the FMD measurement. The power calculation for homocysteine is also included to show that we are able to detect the differences in homocysteine that we expect after betaine and folic acid supplementation.

## FMD

An improvement of about 2 FMD% can be expected, based on previous studies with folic acid (37). The within person standard deviation of the difference in FMD is ~3.6 FMD% with duplicate FMD measurement (38). We need 30 volunteers to find a difference of 1.9 FMD% between folic acid or betaine supplementation relative to placebo (power 80%, alpha 5%). To allow drop-out we will start the study with 40 volunteers.

## Plasma homocysteine

The minimal difference in plasma homocysteine between the folic acid or betaine supplement relative to placebo that we can find statistically significant is about 6.7% (power= 80%, alpha=0.05, SD within homocysteine = 13% (39), SD_delta homocysteine duplo_=(√2 * 13%)/√2 = 13%). This is largely within the expected decrease in plasma homocysteine after folic acid and betaine supplementation of 15-20% (this is 2 umol/L decrease in volunteers with a fasting homocysteine concentration of ~12 umol/L) (32)

# Compensation for volunteers

Volunteers that complete the study will receive € 225,00

If a volunteer decides to discontinue the study, the volunteer will be compensated proportionally. Travel costs will be reimbursed.

# Advantage for participants of the study

This study has no direct advantage for participants of this study. From public health point of view the results of this study can contribute in the future to the assessment whether homocysteine-lowering lowers risk of cardiovascular disease. Increasing the intake of folic acid or betaine might then prove to be healthy because they lower homocysteine.

# Insurance

The Wageningen Centre for Food Sciences has effected an insurance (normal risk) for each volunteers in the study conform the WMO (Wet Medisch wetenschappelijk Onderzoek met mensen). Volunteers will be informed about the insurance.

# Submission personal data to CBP (Dutch Data Protection authority)

The information about this study is submitted to the CBP.

# Appendix I - Flow mediated vasodilation (FMD) measurement

Flow mediated vasodilation (FMD) is a non-invasive measurement in the brachial artery with high resolution ultrasound (5). It is the increase in arterial diameter that is induced by a local increase in arterial blood flow. The increase in arterial blood flow (hyperemia) is induced by inflating a blood pressure cuff around the arm to occlude the arterial flow for 5 min. Release of the blood pressure cuff leads to hyperemia which increases shear stress on endothelial cells. In response to shear stress the endothelial cells produce nitric oxide, a vasodilator. Nitric oxide relaxes the smooth muscle cells in the arterial wall and as a result the diameter of the artery increases. The increase in arterial diameter is measured with high resolution ultrasound.

FMD-Protocol

1. Day before the FMD measurement subjects will receive a standardized diet. Foods are provided by the department of Human Nutrition and Epidemiology
2. Day of the FMD measurement: Subject arrives at the division of Human Nutrition and Epidemiology after an overnight fast (only water is allowed).
3. The subject first rests on a bed for 10-15 min. During this time blood pressure will be measured
4. FMD measurement:
   - Measurement of the basal diameter of the artery in rest (d_basal_)
   - Occlusion of the brachial artery: blood pressure cuff is inflated to a pressure of 200 mmHg for 5 min.
   - Release of blood pressure cuff and measurement of the diameter of the brachial artery for 5 min thereafter (d_maximal_).
5. A blood sample will be taken
6. Breakfast will be served

Calculation of FMD

FMD is calculated as the percentage maximal increase in diameter relative to basal diameter after occlusion:

FMD= {(d_maximal_ - d_basal_) / d_basal_} x 100%

In previous studies of Nicole de Roos the FMD was about 5% (38)

# Appendix II - Blood pressure measurement

Blood pressure protocol

Systolic blood pressure (SBP) and diastolic blood pressure (DBP) will be measured with the DINAMAP PRO 100, according to a standard procedure:

- Subject rests on a bed for 5 min
- Blood pressure is measured on the arm which is not used to measure FMD
- Blood pressure is measured 3 times with 1 min rest in between the measurements
- The mean of the last two blood pressure measurements will be used as study parameter.

# References

1. Ueland, PM, Refsum, H, Beresford, SA, Vollset, SE (2000) The controversy over homocysteine and cardiovascular risk. Am. J. Clin. Nutr. 72: 324-332.

2. Schnyder, G, Roffi, M, Pin, R, Flammer, Y, Lange, H, Eberli, FR, Meier, B, Turi, ZG, Hess, OM (2001) Decreased rate of coronary restenosis after lowering of plasma homocysteine levels. N. Engl. J. Med. 345: 1593-1600.

3. Clarke, R (2000) An overview of the homocysteine lowering clinical trials. In: Homocysteine and vascular disease (Robinson, K., eds.) pp. 413-429. Kluwer Academic Publishers, Dordrecht/Boston/London.

4. Clarke, R & Collins, R (1998) Can dietary supplements with folic acid or vitamin B6 reduce cardiovascular risk? Design of clinical trials to test the homocysteine hypothesis of vascular disease. J. Cardiovasc. Risk. 5: 249-255.

5. Celermajer, DS, Sorensen, KE, Gooch, VM, Spiegelhalter, DJ, Miller, OI, Sullivan, ID, Lloyd, JK, Deanfield, JE (1992) Non-invasive detection of endothelial dysfunction in children and adults at risk of atherosclerosis. Lancet 340: 1111-1115.

6. Takase, B, Uehata, A, Akima, T, Nagai, T, Nishioka, T, Hamabe, A, Satomura, K, Ohsuzu, F, Kurita, A (1998) Endothelium-dependent flow-mediated vasodilation in coronary and brachial arteries in suspected coronary artery disease. Am. J. Cardiol. 82: 1535-1538.

7. Schachinger, V, Britten, MB, Zeiher, AM (2000) Prognostic impact of coronary vasodilator dysfunction on adverse long-term outcome of coronary heart disease. Circulation 101: 1899-1906.

8. Suwaidi, JA, Hamasaki, S, Higano, ST, Nishimura, RA, Holmes, DRJ, Lerman, A (2000) Long-term follow-up of patients with mild coronary artery disease and endothelial dysfunction. Circulation 101: 948-954.

9. Brown, AA & Hu, FB (2001) Dietary modulation of endothelial function: implications for cardiovascular disease. Am. J. Clin. Nutr. 73: 673-686.

10. de Roos, NM, Bots, ML, Katan, MB (2001) Replacement of dietary saturated fatty acids by trans fatty acids lowers serum HDL cholesterol and impairs endothelial function in healthy men and women. Arterioscler. Thromb. Vasc. Biol. 21: 1233-1237.

11. de Roos, NM, Bots, ML, Siebelink, E, Schouten, E, Katan, MB (2001) Flow-mediated vasodilation is not impaired when HDL-cholesterol is lowered by substituting carbohydrates for monounsaturated fat. Br. J. Nutr. 86: 181-188.

12. Lentz, SR, Sobey, CG, Piegors, DJ, Bhopatkar, MY, Faraci, FM, Malinow, MR, Heistad, DD (1996) Vascular dysfunction in monkeys with diet-induced hyperhomocyst(e)inemia. J. Clin. Invest. 98: 24-29.

13. Holven, KB, Holm, T, Aukrust, P, Christensen, B, Kjekshus, J, Andreassen, AK, Gullestad, L, Hagve, TA, Svilaas, A, Ose, L, Nenseter, MS (2001) Effect of folic acid treatment on endothelium-dependent vasodilation and nitric oxide-derived end products in hyperhomocysteinemic subjects. Am. J. Med. 110: 536-542.

14. Tawakol, A, Omland, T, Gerhard, M, Wu, JT, Creager, MA (1997) Hyperhomocyst(e)inemia is associated with impaired endothelium-dependent vasodilation in humans. Circulation 95: 1119-1121.

15. Woo, KS, Chook, P, Lolin, YI, Cheung, AS, Chan, LT, Sun, YY, Sanderson, JE, Metreweli, C, Celermajer, DS (1997) Hyperhomocyst(e)inemia is a risk factor for arterial endothelial dysfunction in humans. Circulation 96: 2542-2544.

16. Schlaich, MP, John, S, Jacobi, J, Lackner, KJ, Schmieder, RE (2000) Mildly elevated homocysteine concentrations impair endothelium dependent vasodilation in hypercholesterolemic patients. Atherosclerosis 153: 383-389.

17. Bellamy, MF, McDowell, IF, Ramsey, MW, Brownlee, M, Bones, C, Newcombe, RG, Lewis, MJ (1998) Hyperhomocysteinemia after an oral methionine load acutely impairs endothelial function in healthy adults. Circulation 98: 1848-1852.

18. Chambers, JC, Obeid, OA, Kooner, JS (1999) Physiological increments in plasma homocysteine induce vascular endothelial dysfunction in normal human subjects. Arterioscler. Thromb. Vasc. Biol. 19: 2922-2927.

19. Usui, M, Matsuoka, H, Miyazaki, H, Ueda, S, Okuda, S, Imaizumi, T (1999) Endothelial dysfunction by acute hyperhomocyst(e)inaemia: restoration by folic acid. Clin. Sci. (Lond. ) 96: 235-239.

20. Hanratty, CG, McGrath, LT, McAuley, DF, Young, IS, Johnston, GD (2001) The effects of oral methionine and homocysteine on endothelial function. Heart 85: 326-330.

21. Verhaar, MC, Wever, RM, Kastelein, JJ, van Dam, T, Koomans, HA, Rabelink, TJ (1998) 5-methyltetrahydrofolate, the active form of folic acid, restores endothelial function in familial hypercholesterolemia. Circulation 97: 237-241.

22. Verhaar, MC, Wever, RM, Kastelein, JJ, van Loon, D, Milstien, S, Koomans, HA, Rabelink, TJ (1999) Effects of oral folic acid supplementation on endothelial function in familial hypercholesterolemia. A randomized placebo-controlled trial. Circulation 100: 335-338.

23. Bellamy, MF, McDowell, IF, Ramsey, MW, Brownlee, M, Newcombe, RG, Lewis, MJ (1999) Oral folate enhances endothelial function in hyperhomocysteinaemic subjects. Eur. J. Clin. Invest. 29: 659-662.

24. Woo, KS, Chook, P, Lolin, YI, Sanderson, JE, Metreweli, C, Celermajer, DS (1999) Folic acid improves arterial endothelial function in adults with hyperhomocystinemia. J. Am. Coll. Cardiol. 34: 2002-2006.

25. Title, LM, Cummings, PM, Giddens, K, Genest, JJJ, Nassar, BA (2000) Effect of folic acid and antioxidant vitamins on endothelial dysfunction in patients with coronary artery disease. J. Am. Coll. Cardiol. 36: 758-765.

26. Doshi, SN, McDowell, IF, Moat, SJ, Lang, D, Newcombe, RG, Kredan, MB, Lewis, MJ, Goodfellow, J (2001) Folate improves endothelial function in coronary artery disease: an effect mediated by reduction of intracellular superoxide? Arterioscler. Thromb. Vasc. Biol. 21: 1196-1202.

27. Doshi, SN, McDowell, IF, Moat, SJ, Payne, N, Durrant, HJ, Lewis, MJ, Goodfellow, J (2002) Folic acid improves endothelial function in coronary artery disease via mechanisms largely independent of homocysteine lowering. Circulation JID - 0147763 105: 22-26.

28. Thambyrajah, J, Landray, MJ, McGlynn, FJ, Jones, HJ, Wheeler, DC, Townend, JN (2000) Does folic acid decrease plasma homocysteine and improve endothelial function in patients with predialysis renal failure? Circulation 102: 871-875.

29. Pullin, CH, Ashfield-Watt, PA, Burr, ML, Clark, ZE, Lewis, MJ, Moat, SJ, Newcombe, RG, Powers, HJ, Whiting, JM, McDowell, IF (2001) Optimization of dietary folate or low-dose folic acid supplements lower homocysteine but do not enhance endothelial function in healthy adults, irrespective of the methylenetetrahydrofolate reductase (C677T) genotype. J. Am. Coll. Cardiol. 38: 1799-1805.

30. van Dijk, RA, Rauwerda, JA, Steyn, M, Twisk, JW, Stehouwer, CD (2001) Long-term homocysteine-lowering treatment with folic acid plus pyridoxine is associated with decreased blood pressure but not with improved brachial artery endothelium-dependent vasodilation or carotid artery stiffness: a 2-year, randomized, placebo-controlled trial. Arterioscler. Thromb. Vasc. Biol. 21: 2072-2079.

31. Wilmink, HW, Stroes, ES, Erkelens, WD, Gerritsen, WB, Wever, R, Banga, JD, Rabelink, TJ (2000) Influence of folic acid on postprandial endothelial dysfunction. Arterioscler. Thromb. Vasc. Biol. 20: 185-188.

32. Steenge, G.R., Verhoef, P., Katan, M.B. (2002) Effect of betaine on plasma concentrations of fasting and post-methionine loading homocysteine in healthy volunteers. unpublished work

33. Brouwer, IA, Verhoef, P, Urgert, R (2000) Betaine supplementation and plasma homocysteine in healthy volunteers. Arch. Intern. Med. 160: 2546-2547.

34. Kerins, DM, Koury, MJ, Capdevila, A, Rana, S, Wagner, C (2001) Plasma S-adenosylhomocysteine is a more sensitive indicator of cardiovascular disease than plasma homocysteine. Am. J. Clin. Nutr. 74: 723-729.

35. Boger, RH, Lentz, SR, Bode-Boger, SM, Knapp, HR, Haynes, WG (2001) Elevation of asymmetrical dimethylarginine may mediate endothelial dysfunction during experimental hyperhomocyst(e)inaemia in humans. Clin. Sci. (Lond. ) 100: 161-167.

36. Stuhlinger, MC, Tsao, PS, Her, JH, Kimoto, M, Balint, RF, Cooke, JP (2001) Homocysteine impairs the nitric oxide synthase pathway: role of asymmetric dimethylarginine. Circulation 104: 2569-2575.

37. Chambers, JC, Ueland, PM, Obeid, OA, Wrigley, J, Refsum, H, Kooner, JS (2000) Improved vascular endothelial function after oral B vitamins: An effect mediated through reduced concentrations of free plasma homocysteine. Circulation 102: 2479-2483.

38. de Roos, NM (2001) Trans fatty acids, HDL-cholesterol, and cardiovascular disease risk. Effects of dietary changes on vascular reactivity. Thesis/Dissertation. Wageningen University.

39. Voortman, A, Melse-Boonstra, A, Schulz, JM, Burema, J, Katan, MB, Verhoef, P (2001) Optimal time interval between repeated blood sampling for measurements of total homocysteine in healthy individuals. Clin. Chem. 47: 1839-1841.
