# Supplementary material for: Effect of Homocysteine-Lowering Nutrients on Blood Lipids: Results from Four Randomised, Placebo-Controlled Studies in Healthy Humans
Source: PLoS Med. 2005 May 31;2(5):e135. doi: 10.1371/journal.pmed.0020135 (PMC1140947; doi:10.1371/journal.pmed.0020135)
Supplement: Protocol S2 — (840 KB PDF). [file pmed.0020135.sd002.pdf]

TNO Nutrition and Food Research

Nederlandse Organisatie voor
toegepast-natuurwetenschappelijk
onderzoek / Netherlands Organisation
for Applied Scientific Research

Nutritional Physiology
Location Zeist
Utrechtseweg 48
P.O. Box 360
3700 AJ Zeist
The Netherlands

www.tno.nl

P +31 30 694 41 44
F +31 30 695 72 24
infofood@voeding.tno.nl

TNO Protocol

P4963 | Revised final |

Effect of choline on post-methionine loading and fasting concentrations of plasma homocysteine in healthy volunteers

Date	19th March, 2003
At the request of	Wageningen Centre for Food Sciences
Drafted by	Dr ir E.J. Brink
TNO project number	010.21110
TNO study code	4963
Status	Revised final
Previous versions	Draft 01, 15th January 2003<br>Draft 02, 20th February 2003<br>Final, 3th March 2003
Number of pages	50
Number of appendices	8 Appendices<br>18 Forms

All rights reserved.
No part of this publication may be reproduced and/or published by print, photoprint, microfilm or any other means without the previous written consent of TNO.

In case this report was drafted on instructions, the rights and obligations of contracting parties are subject to either the Standard Conditions for Research Instructions given to TNO, or the relevant agreement concluded between the contracting parties. Submitting the report for inspection to parties who have a direct interest is permitted.

© 2003TNO

Contents

1 List of Abbreviations (alphabetical order) 5

2 Synopsis 6

3 Schedule of assessments 10

4 Responsible personnel and test facilities 11
4.1 Sponsor(s) 11
4.2 Testing facilities and responsible personnel 11
4.3 Responsibilities 13

5 Introduction 15

6 Objective of the study 17

7 Study design 18

8 Study substances and study treatments 19
8.1 Study substances 19
8.1.1 Description 19
8.1.2 Supply to TNO 19
8.1.3 Storage conditions 19
8.1.4 Chemical analyses 19
8.1.5 Remainder of study substances 19
8.2 Study treatments 19
8.2.1 Description 19
8.2.2 Blinding and unblinding 20
8.2.3 Supply to subjects 21
8.2.4 Labelling 21
8.3 Accountability 21
8.4 Safety of treatment 22
8.5 Standardized diet 23
8.6 Compliance with study instructions and treatment 23

9 Subjects 24
9.1 Population base 24
9.2 Number of subjects and rationale for the number 24
9.3 Replacement 24
9.4 Inlusion criteria 24
9.5 Exclusion criteria 25

10 Informing the subjects26

11 Study parameters 27
11.1 Pre-study screening 27
11.1.1 Medical History 27
11.1.2 Physical examination 27
11.1.3 Clinical laboratory test 27
11.1.4 Total homocysteine and B-vitamins 27

11.2 In-study 27
11.2.1 Additional parameters 28
11.2.2 Adverse events 28

12 Study procedures 29
12.1 General 29
12.2 Pre-study screen, eligibility and selection 29
12.3 Allocation to entry number and study treatment 30
12.4 Assessments 30
12.5 Compliance 31
12.6 Adverse Events (AEs) 31
12.7 Serious Adverse Events (SAEs) 31
12.8 Safety 31
12.9 Post-study screen 32
12.10 Criteria for withdrawal or premature discontinuation 32

13 Code and handling of samples 33
13.1 Sample coding 33
13.2 Sample collection, preparation and storage 33
13.2.1 Sample collection pre-study 33
13.2.2 Sample collection in-study 34
13.3 Remainder samples and archive samples 36

14 Documentation 37

15 Data management and statistics 38
15.1 Data management 38
15.2 Missing values 38
15.3 Statistical analysis 38
15.3.1 Descriptive parameters 38
15.3.2 Derived parameters 38
15.3.3 Analysis 38

16 Reporting 40

17 Publication 41

18 Timing 42

19 Insurance of subjects 43

20 Ethics and quality 44
20.1 General 44
20.2 Good Clinical Practice 44
20.3 Quality assurance and monitoring 44

21 Retention of records, samples and specimens 45

22 References 46

23 Approval of the protocol 48
23.1 Sponsor 48

23.2 TNO Nutrition and Food Research 48

24 List of Appendices 49

25 List of forms 50

1 List of Abbreviations (alphabetical order)

AE : Adverse Event
ALAT : Alanine Amino Transferase
ALP : Alkaline phosphatase
ANOVA: Analysis of Variance
ASAT : Aspartate-amino Transferase
BMI : Body Mass Index
BP : Blood Pressure
CCMO : Central Committee on Research involving Human Subjects
CRO : Contract Research Organisation
GCP : Good Clinical Practice
GLP : Good Laboratory Practice
GMP : Good Manufacturing Practice
γ -GT : Gamma Glutamyl Transferase
Hb : Haemoglobin
HDL : High Density Lipoprotein
HR : Heart rate
Ht : Haematocrit
ICH : International Conference on Harmonisation of Technical Requirements for Registration of Pharmaceuticals for Human Use
LDL : Low Density Lipoprotein
METC : Medisch Ethische Toetsings Commissie (Medical Ethics Committee / Institutional Review Board)
OECD : Organisation for Economic Cooperation and Development
QAU : Quality Assurance Unit
RBC : Red Blood Cell
SAE : Serious Adverse Event
SAH : S-adenosylhomocysteine
SAM : S-adenosylmethionine
SOP : Standard Operating Procedure
TNO : Nederlandse organisatie voor Toegepast Natuurwetenschappelijk Onderzoek (Netherlands Organization for Applied Scientific Research)
VLDL : Very Low Density Lipoprotein
VTC : Visit Time Code
WBC : White Blood Cell
WCFS : Wageningen Centre for Food Sciences
WHO : World Health Organisation

2 Synopsis

Title: Effect of choline on post-methionine loading and fasting concentrations of plasma homocysteine in healthy volunteers

Sponsor
- Name : Wageningen Centre for Food Sciences
- Study code : -

CRO
- Name : TNO Nutrition and Food Research (TNO)
- Study code : 4963

Design : Randomized, cross-over, placebo controlled, double-blind

Objectives :
- To investigate the effect of daily intake of about 2.5 g choline on fasting plasma homocysteine
- To investigate the effect of daily intake of about 2.5 g choline on post-methionine loading plasma homocysteine
- To investigate the effect of single intake of about 1.25 g choline on post-methionine loading plasma homocysteine

Participants
- Description: Apparently healthy male volunteers
- Number: 26
- Inclusion criteria:
  - Healthy males as assessed by the health and lifestyle questionnaire, physical examination and results of the pre-study laboratory tests
  - Age at day 01 of the study: 50-80 years
  - Body Mass Index (BMI) ≤ 33 kg/m^2
  - Normal Dutch eating habits, including use of breakfast
  - Willing not to use supplements containing B-vitamins, lecithin, choline (derivatives) or betaine from the oral information session until the end of the study
  - Voluntary participation
  - Having given their written informed consent
  - Willing to comply with the study procedures, including dietary restrictions
  - Willing to accept use of all nameless data, including publication, and the confidential use and storage of all data
  - Willing to accept the disclosure of the financial benefit of participation in the study to the authorities concerned
- Exclusion criteria:
  - Participation in any clinical trial including blood sampling and/or administration of products up to 90 days before Day 01 of this study
  - Participation in any non-invasive clinical trial up to 30 days before Day 01 of this study
  - Having a history of medical or surgical events that may significantly affect the study outcome, including cardiovascular disease or hypertension
  - Use of medication known to interfere with homocysteine metabolism

- Plasma total homocysteine concentrations > 26 µmol/L
- Plasma vitamin B6 concentrations ≤ 15 nmol/L
- Serum vitamin B12 concentrations < 138 pmol/L
- Serum folic acid concentrations < 5.0 nmol/L
- Alcohol consumption > 28 units/week
- Reported unexplained weight loss or gain of > 2 kg in the month prior to the pre-study screening
- Reported slimming or medically prescribed diet
- Reported food allergy
- Reported vegan or macrobiotic
- Use of B-vitamin supplements, lecithin, or supplements containing choline (derivatives) or betaine, more than once weekly < 1 month before screening
- Recent blood or plasma donation (< 1 month prior to the start of the study)
- Not willing to stop blood or plasma donation during the study
- Personnel of TNO Nutrition and Food Research, their partner and their relatives in the first and second remove
- Not having a general practitioner
- Not willing to accept information-transfer concerning participation in the study, or information regarding his/her health, like laboratory results, findings at anamnesis or physical examination and eventual adverse events to and from his general practitioner

NOTE: plasma homocysteine concentration will be used as selection parameter. From the eligible subjects, males with the highest homocysteine concentrations (but not higher than 26 umol/L) will be selected. For this purpose we aim at having approximately 40 eligible subjects.

Study substances
- Test substance : Phosphatidylcholine
- Reference substance : Cooking oil mainly consisting of diacylglycerol

Study treatments
- Choline : about 2.5 g/d choline from phosphatidylcholine (this corresponds with about 30 g/d phosphatidylcholine)
- Reference : about 30 g/d reference

Study substances will be consumed divided over the day, two times per day (breakfast, and dinner). Half of the dose will be given each time (each dose mixed in about 200 ml custard). Treatments will be separated by a wash-out period of 2 wk.

<u>Methionine loading test:</u>
On day 01 and 15 of each treatment a methionine loading test will be performed. All subjects ingest methionine (100 mg/kg body weight). Methionine will be given with a normal breakfast. Methionine-rich products will be avoided. Blood samples will be collected before loading 0h (fasting) and at 6h after loading (non-fasting).

Study period
- Duration : 6 wk (two periods of 2 wk with wash-out of 2 wk in between)
- Lodging : Not applicable
- Visits to TNO : 2 times pre-study screening and 8 times during the study

Restrictions

- Life-style: Stay in metabolic ward for approximately 6-7 h after each methionine load (4 times)
- Diet :
  - Continue habitual intake of coffee and tea during the study
  - Consumption of coffee is restricted to one cup at breakfast and one cup during the morning at the days of the methionine loading test
  - No consumption of liver, spinach and shrimps
  - Restricted consumption of liver products (2x per week)
  - No consumption of eggs and egg-rich products
  - Restricted consumption of egg-containing products
  - Restricted consumption of products containing choline

Dietary restrictions start 2 weeks before the start of the study

Tests at screening

- Anamnesis : Lifestyle and health questionnaire
- Physical examination : Body weight, height, vital signs
- Clinical laboratory (all fasting):
  - Haematology (RBC, WBC, platelets, Ht, Hb, differential white blood cell count)
  - Serum clinical chemical profile (gamma-GT, ALAT, ASAT, ALP, glucose, total bilirubin, albumin, creatinine, urea, sodium, potassium, chloride)
  - Dipstick urinalysis (protein, glucose, leucocytes, erythrocytes, nitrite, pH, ketones, bilirubin, urobilinogen). If the dipstick test gives values above the normal range for leucocytes, blood or protein, a microscopic inspection of sediment of urine will be done.
- Other blood analysis (fasting)
  - Total homocysteine and vitamin B6 in plasma
  - Vitamin B12 and folic acid in serum

Test parameters in study

Blood analysis

- Total homocysteine will be measured in fasting plasma samples on days 01, 13, 15, 29, 41, 43 and in non-fasting plasma samples collected at 6h after methionine loading on day 01, 15, 29 and 43
- Total cholesterol, HDL cholesterol, LDL-cholesterol (calculated), triacylglycerols in fasting serum samples on day 13, 15, 41 and 43
- gamma-GT, ALAT, ASAT, ALP, creatinine in fasting serum samples on days 13, 15, 41 and 43
- Plasma vitamin B6, and serum vitamin B12 and folic acid in fasting samples on days 13, 15, 41 and 43
- Betaine, dimethylglycine and choline in fasting and non-fasting plasma samples collected at 6h after methionine loading on day 01, 15, 29, 43

Body weight

- Body weight will be measured on day 01, 15, 29, 43

Compliance

All subjects will report their intake of study substances and deviations from dietary restrictions

Additional parameters
On all blood sampling occasions samples will be stored for possible later analysis of methionine (and other amino acids), S-adenosylmethionine (SAM), S-adenosylhomocysteine (SAH), choline and betaine metabolites and cholesterol derivatives. Additional on day 13 blood will be stored for possible later analysis of polymorphisms associated with homocysteine metabolism, folate metabolism and/or choline metabolism.

Adverse events
Well-being questionnaire will be filled-in by the subjects every visit

Tests post-study
Interview with the (assistant) Medical Investigator

Sample collection
- Blood sampling: Pre-study (clinical laboratory, safety parameters)
  In study day 01, 13, 15, 29, 41, 43
- Urine collection: Pre-study (spot urine for urine safety parameters)

Statistical analysis
- Descriptive : Subject characteristics, adverse events, compliance
- ANOVA: Analysis will be carried out with subjects and treatment as factors on body weight, fasting plasma homocysteine, increase in plasma homocysteine (6h) after methionine loading after single dose, increase in plasma homocysteine (6h) after methionine loading after 14d consumption, blood lipids, liver enzymes and creatinine, vitamin B6, B12, folate, betaine, dimethylglycine and choline

Responsibilities

- Protocol writing	: Department of Nutritional Physiology, TNO
- Insurance subjects for WMO	: TNO Nutrition and Food Research for WCFS
- Supply study substances	: Department of Nutritional Physiology, TNO
- Analysis study substances	: WCFS
- Laboratory analyses	
- Clin Lab, pre-study	: Department of General Toxicology, TNO
- Blood lipids and liver enzymes	: Department of General Toxicology, TNO
- Homocysteine	: Wageningen University and Research Centre
- Vit B6, B12, Folate	: Department of Food Analysis, TNO
- Betaine, dimethylglycine, choline	: WCFS
- Statistical analysis	: Department of Nutritional Epidemiology, TNO
- Drafting report	: Department of Nutritional Physiology, TNO
- Quality assurance	: Quality Assurance Unit of TNO

Tentative time schedule

- Protocol writing	: November 2002 – February 2003
- Signed quotation for total study	: End February 2003
- Approval of METC	: End March 2003
- Recruitment and selection	: April/May 2003
- Study initiation	: End May 2003
- Completion clinical part	: Mid July 2003
- Completion lab analysis	: End August 2003
- Completion statistical analysis	: Mid October 2003
- Draft unaudited TNO Report	: Mid November 2003

3 Schedule of assessments

	Study day*									
Visit	Pre-study		1	2	3	4	5	6	7	8
Activity	Pre study **	-14	01	05/ 06¥	13	15	29	35	41	43
Anamnesis	+									
Physical examination	+									
Body weight	+		+			+	+			+
Urine sampling	+									
Start dietary restrictions; to be continued throughout the study		+								
Blood sampling (fasting)	+		+		+	+	+		+	+
Blood sampling (non-fasting)			+			+	+			+
Methionine loading test			+			+	+			+
Well-being questionnaire			+	+	+	+	+	+	+	+
Dispense study substances			+	+			+	+		
Dispense one portion study substance at breakfast			+		+	+	+		+	+
Return empty bowls				+		+		+		+
Compliance check				+		+		+		+
End of trial/Post study screen										+

** Maximal 8 weeks before day 01 of the study

The study will have a staggered start in two groups.

* For each staring group Day 01 is defined as the first day of the clinical part of the study

¥ For the first starting group this visit will be on day 06 of the study, for the second starting groups this visit will be on day 05 of the study

4 Responsible personnel and test facilities

4.1 Sponsor(s)

Wageningen Centre for Food Sciences (WCFS)
P.O.Box 557
6700 AN Wageningen
The Netherlands

Responsible persons

Location
Wageningen University & Research Centre
Division of Human Nutrition and Epidemiology
Bomenweg 2, P.O.Box 8129
6700 EV Wageningen, The Netherlands

Project Manager WCFS
Ms. P. Verhoef, PhD
Phone : +31 317 48 20 77
Fax : +31 317 48 33 42
E-Mail : petra.verhoef@wur.nl

Study monitor
Ms. M. Olthof, PhD
Phone : +31 317 48 43 36
Fax : +31 317 48 33 42
E-mail : margreet.olthof@wur.nl

4.2 Testing facilities and responsible personnel

TNO Nutrition and Food Research
Department of Nutritional Physiology
Utrechtseweg 48
P.O. Box 360
3700 AJ ZEIST
Phone : +31 30 69 44 144
Fax : +31 30 69 57 224

Wageningen University & Research Centre
Division of Human Nutrition and Epidemiology
Bomenweg 2
P.O.Box 8129
6700 EV Wageningen
The Netherlands
Phone : +31 317 250 91 11
Fax : +31 317 254 14 74

Responsible persons at TNO:

Department of Nutritional Physiology

Principal Investigator
Ms. E.J. Brink, PhD
Phone : 030 694 47 61
Fax : 030 694 49 28
E-mail : L.Brink@voeding.tno.nl

Deputy Principal Investigator
Ms. T. van Vliet, PhD
Phone : 030 694 49 69
Fax : 030 694 49 28
E-mail : T.vanVliet@voeding.tno.nl

Medical Investigator
Ms. W.A.A. Klöpping-Ketelaars, MD, PhD
Phone : 030 694 46 22
Fax : 030 694 49 28
E-mail : Klopping@voeding.tno.nl

Deputy Medical Investigator
Mr. H. Viersen, MD,
Phone : 030 694 46 64
Fax : 030 694 49 28
E-mail : Viersen@voeding.tno.nl

Senior Research Nurse
Mr. F.W. Sieling
Phone : 030 694 49 76
Fax : 030 694 49 28
E-mail : Sieling@voeding.tno.nl

Department of Nutritional Epidemiology

Senior Dietitian
Ms. S. Westenbrink
Phone : 030 694 47 50
Fax : 030 695 79 52
E-mail : Westenbrink@voeding.tno.nl

Statistician
Mr. C. Kistemaker, BSc
Phone : 030 694 47 57
Fax : 030 695 79 52
E-mail : Kistemaker@voeding.tno.nl

Department of Food and Food Supplement Analysis

Mr. W.H.J Vaes, PhD
Phone : 030 694 40 78
Fax : 030 695 67 42
E-mail : Vaes@voeding.tno.nl

Department of General Toxicology

Clinical Chemical Laboratory
Mr. J.F. Catsburg
Phone : 030 694 45 01
Fax : 030 696 02 64
E-mail : Catsburg@voeding.tno.nl

Responsible person at Wageningen UR:

Investigator
Mr. P.J.M. Hulshof, MSc
Phone : 0317 484 824
Fax : 0317 483 342
E-mail : paul.hulshof@wur.nl

4.3 Responsibilities

The sponsor is responsible for the financial compensation for the conduct of the study and analysis of fatty acid composition and choline content of the study substances. In addition the sponsor is responsible for analysis of betaine, choline and dimethylglycine in plasma samples obtained during the clinical phase. Moreover the sponsor is responsible for the insurance according to the "WMO". At request of the sponsor TNO insures the volunteers according to the WMO. Insurances for material damage and accidents during the travel to and from TNO and during the stay at TNO are TNO's responsibility.

Ms. Brink will be responsible for the overall conduct of the study.

Ms. van Vliet will be responsible for the overall conduct of the study in case Ms. Brink is not available.

Ms. Klöpping-Ketelaars will be responsible for the selection of the subjects, medical aspects of the study, documentation, and interpretation and reporting of the AEs and SAEs.

Mr. Viersen will be responsible for the selection of the subjects, medical aspects of the study, documentation, and interpretation and reporting of the AEs and SAEs in case Ms. Klöpping-Ketelaars is not available.

Mr. F.W. Sieling will be responsible for the daily conduct of the clinical part of the study, the dispense of the study substances to the subjects and the contacts with the subjects.

Ms. Westenbrink will be responsible for receipt, coding, labelling, distribution, storage, accountability of study substances and for preparation of the portions custard containing the study substances.

Mr. C. Kistemaker will be responsible for randomization of the subjects, for the allocation of the subjects to entry-numbers and to treatment, for data management and for the conduct of the statistical analysis.

Mr. J. F. Catsburg will be responsible for the conduct of the pre-study clinical tests in blood and urine and for analysis of blood lipids and liver enzymes during the clinical phase.

Mr. W.H.J. Vaes will be responsible for the conduct of the analyses of vitamin B6 in plasma and vitamin B12, and folate in serum.

Mr. P. Hulshof will be responsible for the conduct of the homocysteine analyses in plasma.

5 Introduction

A high homocysteine concentration in blood is a potential risk factor for cardiovascular disease: a decrease in homocysteine of 5 umol/L lowers risk of cardiovascular disease up to 20-30% (1). Homocysteine is formed through demethylation of methionine, which mainly occurs in the liver. Homocysteine can be broken down through the transsulfuration pathway, or it can be remethylated to methionine. The methyl group necessary for remethylation can be derived from 5-methyltetrahydrofolate or from betaine (Fig. 1). It is known that intake of extra folic acid decreases plasma homocysteine in healthy volunteers (2, 3), and additional intake of betaine lowers plasma homocysteine in hyperhomocysteinemic subjects (4, 5). Recently we found that betaine also lowers homocysteine in healthy volunteers (6-8). Betaine can be derived from the diet, and it can be synthesized in the body through oxidation of choline. Choline is present in considerable amounts in foods, but the effects of choline on plasma homocysteine are unknown. Therefore we will investigate the effects of ingestion of choline on plasma homocysteine concentrations in healthy volunteers.

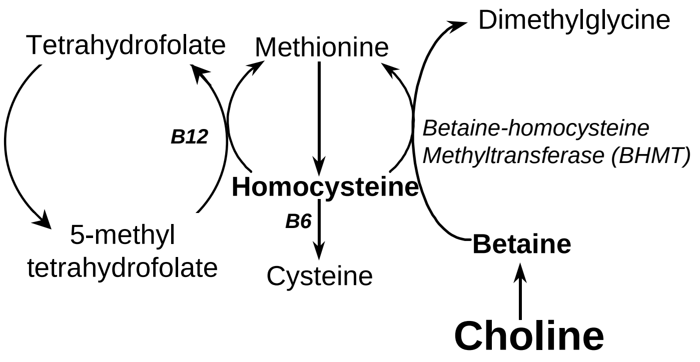

Figure 1. Homocysteine metabolism

Choline in foods

Choline is mainly present in foods in the form of phosphatidylcholine (lecithin) (9). Choline-rich food sources are generally foods that are also high in fat, such as milk, liver, eggs, and peanuts. With the current trend to reduce the intake of high-fat foods the intake of choline might also be reduced. Especially for people with a low-fat intake it is important that the intake of choline remains sufficient, since choline is an indispensable nutrient for humans. There are few data on amounts of betaine and choline (derivatives) in foods, but prof. Steven Zeisel (University of North Carolina at Chapel Hill, USA) is currently measuring these compounds in foods. Dietary reference intakes have been defined for choline: 425 mg/d choline for women and 550 mg/d for men (10). The estimated daily intake of choline is between 300-1000 mg/day. Choline is not considered an essential nutrient because it can be synthesized in the body via methylation of phosphatidyletholamine. However, *de novo* synthesis of choline is not always sufficient to meet the human requirements for choline. When healthy males with normal folate and vitamin B12 status are fed a choline-deficient diet their plasma choline and phosphatidylcholine concentrations decrease and they develop liver damage (increased alanine aminotransferase concentrations) (11). Humans fed with a total

parenteral nutrition with adequate methionine and folate, but without choline, develop fatty liver and liver damage. This effect can be reversed by adding choline to the parenteral nutrition (12, 13). This emphasises the importance of the diet as a source of choline.

Functions of choline in the body
Choline has many functions in the body, mainly in brain function and development, liver function, and it is a source of labile methyl groups (14). Choline is a precursor for the neurotransmitter acetylcholine and for phosphatidylcholine, a structural component of all cell membranes. In the liver, choline is required for secretion of Very Low Density Lipoprotein (VLDL) and essential for normal lipid-cholesterol transport and metabolism. Choline is a source of labile methyl groups that can be made available through oxidation of choline into betaine. This irreversible conversion mainly occurs in the liver and kidney. Methyl groups are important for DNA methylation. Hypomethylation of DNA during choline deficiency might be related to carcinogenesis. Furthermore, betaine donates its methyl groups to homocysteine to form methionine and subsequently lower plasma homocysteine.

Reason for the study
There is no information on the effect of choline intake on plasma homocysteine in healthy humans. Therefore the objective of this study is to investigate the effect of daily ingestion of about 2.5 gram choline on plasma homocysteine concentrations in healthy humans. We decided to choose the highest achievable dose. The current tolerable upper intake level of choline is 3.5 gram/day for adults (10). Assuming a 50% oxidation of choline into betaine it is expected that a daily ingestion of 2.5 g choline has a homocysteine lowering potential. In our previous study (3818) daily ingestion of 1.5 g betaine has shown to be effective in homocysteine lowering. We will supply choline in the form of phosphatidylcholine (lecithin) because this is the major form of choline in foods. Ingestion of phosphatidylcholine also has a larger effect on plasma choline concentrations than equivalent amount of choline chloride and furthermore phosphatidylcholine has fewer side effects (see section 8.4) than choline chloride (18, 22). To provide about 2.5 g choline, about 30 g phosphatidylcholine (60%-70% pure) should be consumed. For phosphatidylcholine (lecithin) no upper intake levels are defined. It has been indicated that dosages up to 40 g are tolerated (see section 8.4).

6 Objective of the study

The objectives of the present study are

- To investigate the effect of daily intake of about 2.5 g choline on fasting plasma homocysteine
- To investigate the effect of daily intake of about 2.5 g choline on post-methionine loading plasma homocysteine
- To investigate the effect of single intake of about 1.25 g choline on post-methionine loading plasma homocysteine

7 Study design

The study is designed as a randomized, cross-over, placebo-controlled, double-blind intervention trial in free living volunteers

8 Study substances and study treatments

8.1 Study substances

8.1.1 Description

- Test substance : Phosphatidylcholine (Phoschol©, American Lecithin Company, Oxford, UK)
- Reference : Cooking oil consisting mainly of diacylglycerol (Enova™, Archer Daniels Midland Company, Filderstadt, Germany)

Methionine loading test : L-methionine (Methionimum apyrogeen, BUFA B.V., Pharmaceutical products)

8.1.2 Supply to TNO

Study substances are commercially available products and will be obtained directly from the different companies including relevant documentation on the composition and safety of the study substances. The composition available at this moment is given in P4963 B08 and section 8.4. A dietician not involved in the study will attribute codes “A” or “B” to the test and reference substance.

8.1.3 Storage conditions

The study substances will be stored at room temperature at a dry place.

8.1.4 Chemical analyses

The study substances will be analysed for choline concentration and fatty acid distribution before the start of the clinical part under responsibility of WCFS. WCFS will supply the Principal Investigator with results of these analysis at least two weeks before the start of the study.

8.1.5 Remainder of study substances

Most of the remaining study substances will be discarded at the end of the clinical part of the study after approval by the principal investigator. Taking in account the possible need for additional analyses, part of the remaining study substances will be stored for 2 years after the end of the clinical part of the study if the nature of the substance allows it.

8.2 Study treatments

8.2.1 Description

Test treatment : about 2.5 g/d choline during 2 wk (corresponds with consumption of about 30g/d test substance during 2 wk)
Reference treatment : about 30 g/d reference substance during 2 wks

Study substances will be consumed divided over the day, two times per day (breakfast and dinner). Half of the dose will be given each time (each dose mixed in about 200 ml custard).

Treatments will be separated by a wash-out period of 2 wk.

Individual portions of custard mixed with the study substances will be prepared by the dietician not further involved in the conduct of the study according to a written instruction that will be authorized by the Principal Investigator. The individual portions will be labeled as indicated in section 8.2.4.
The test substance contains licorice flavoring. To guarantee double-blind conduct of the study the same type of flavoring has to be used for the reference treatment. The exact type of flavour and the amount to be added is under investigation now and will be available before the start of the study. This will be described in an amendment to the protocol.

Methionine will be packed in small brown bottles in individually labelled portions (100 mg/kg body weight) on basis of the pre-study body weight (P4963 F13). Label coding is described in section 8.2.4.

8.2.2 Blinding and unblinding

All treatments will be blinded for the:
1. Subjects
2. Sponsor
3. Principal investigator, medical investigator and their staff involved in the conduct of the study
4. Members of the Medical Ethics Committee of TNO (TNO-METC)
5. Inspectors involved in the inspection of the clinical part of the study

Only the person who attributed the codes “A” or “B” to the test and reference substance will know the meaning of the codes and will supply the principal investigator at TNO with a sealed envelope containing the key of the codes.

Before the start of the clinical part of the study, the statistician will inform the dietician and the senior research nurse in writing about the allocation to entry number.

Before the clinical part of the study, the statistician will inform the dietitian responsible for labelling of the study substances, in writing of the treatment allocation of the subjects (pre-entry number, entry number and treatment).

The statistician will supply the principal investigator with sealed envelopes per subject, containing the treatment allocation. The principal investigator informs the medical investigator about the place of storage of, and access to these envelopes. During the study, only medical urgency will allow the medical investigator to open an envelope for breaking the code only for the subjects needed. When an envelope is opened, date, time and reason will be documented.

The statistician will unblind the study after the database is closed and after the
1. AEs have been interpreted and reported by the medical investigator to the principal investigator,
2. Laboratory reports on the analyses of the study parameters have been authorized,
3. Decisions on subject or data exclusions have been made.

8.2.3 Supply to subjects

On days 01, 05 or 06, 29 and 35 subjects will be provided with a labelled bag containing tightly closed small bowls containing about 200 mL custard mixed with half of the daily dose of the study substance. When the second visit is on day 05, the number of portions dispensed on day 01 will be 8; when the second visit is on day 06 the number of portions dispensed on day 01 will be 10. The number of portions dispensed on day 05 will be 18 or on day 06 will be 16. The number of portions dispensed on day 29 will be 12 and on day 35 will be 14. On days 01, 13, 15, 29, 41 and 43 one portion will be supplied and consumed at TNO. Dispense of the study substances will be registered on form P4963 F03. On days 05 or 06, 15, 35 and 43 the unused study substances (if any) will be returned to TNO. This will be registered on form P4963 F03 and on form GEN/022 F02. Shelf life will be confirmed before the start of the study. If necessary the dispense schedule will be adapted. Such a change will be described in an amendment.

Subjects will receive instructions for consumption of the portions and for storage of the study substances. Daily intake will be registered on form P4963 F06b.

8.2.4 Labelling

Study substances will be labelled according to a study specific instruction as described in SOP DHDV/ALG/711.

Each small bowl with the study substance will be labelled by a preprinted label, as follows:

First line: TNO study number (4963) followed by a slash, followed by the 2-digit entry number, followed by a slash, followed by a day number.
Second line: portion code (“portie 1” or “portie 2”)

e.g. 4963/01/01
portie 1

Furthermore, each small bowl containing study substance will be labelled with the following text:

“TNO Voeding, studie 4963
Vla met 15 g phosphatidylcholine of diacylglycerol-olie
Bewaren in de koelkast”

Each brown plastic bottle containing methionine will be labelled with a preprinted label, as follows:
First line : methionine
Second line : TNO study number (4963) followed by a slash, followed by the 2-digit entry number, followed by a slash, followed by a ‘d’ and day number

e.g. methionine
4963/01/d01

8.3 Accountability

Accountability of the study products will be done according to SOP DHDV/ALG/710. The study products will be managed by the dietician. Receipt of the study products and registration of storage by the dietician will be recorded on forms DHDV/ALG/710 F01

and F02. Dispense of study products to the subjects and return by the subjects will be registered on P4963 F03.

8.4 Safety of treatment

Choline is a dietary component. Estimates of intake of choline from food or food supplements vary between 300 and 1000 mg/day. Dietary reference intakes for choline have been defined as 425 mg/day for women and 550 mg/day for men (10).

The critical adverse effect from high choline intake is hypotension, with corraborative evidence on cholinergic side effects (e.g. sweating, vomiting, diarrhea) and fishy body odour (15-19). These symptoms are found with therapeutic doses higher than 10 gram/day of choline chloride. Fishy body odour results from excretion of excessive amounts of trimethylamines, a choline metabolite. Intake of lecithin does not result in fishy body odour because it generates little methylamines (18). Mild hepatotoxicity was reported in patients receiving 3 gram/day of choline magnesium trisalicylate, but salicylate was probably causing hepatotoxicity, rather than choline (20). Humans treated with 6 g/d of choline chloride had no liver toxicity (21). Individuals with trimethylaminuria (fish odour syndrome), renal disease, liver disease, depression and Parkinson’s disease may have increased susceptibility to the adverse effects of choline. The current tolerable upper intake level is 3.5 gram/day for adults (10). The dosage used in the present study is about 2.5 g/d.

Choline is mainly present in foods in the form of phosphatidylcholine (lecithin). Lecithin is generally recognized as safe (GRAS). No upper intake levels are defined. In a review of Fiume (23) it is indicated that up to 100 g/d of lecithine has been administered to individuals without toxic effects and 25 g/d was generally well tolerated. Johnson et al. (24) stated that dosages of 40 g are tolerated by most individuals. Higher dosages may result in abdominal bloating, diarrhea and vomiting.
The dosage used in the present study is about 30 g/day. Based on this information no major problems with respect to tolerance are expected. Phosphatidylcholine is a fat and thus metabolized as such. A dosage of 30 g fat per day has been applied in several clinical trials without adverse effects. To minimise possible adverse events the dosage will be administered divided over 2 portions during the day.

No drug interactions with phosphatidylcholine (lecithin) or choline are known.

Diacylglycerol (DAG) is a component present in edible fats and is GRAS. DAG oils were used in an intervention trial in dosages providing about 8 g DAG during 12 weeks (25) or 16 weeks (26) without reported adverse effects. No information is available on human exposure of higher doses of DAG. In a two-year chronic study in rats no toxicologically significant or treatment related effects of diacylglycerol oil consumption at levels up to 5,3% in the diet were observed (27). This corresponds with about 12 g/kg body weight per day for rats. For a man of 70 kg this means a daily intake of 840 g/day. A consumption of 30 g per day as proposed in the present study results in a margin of safety of 28.

Both phosphatidylcholine (lecithin) and diacylglycerol are fatty substances. As such the dosages given in the present study will provide an additional energy intake of about 260 kcal per day. For each treatment period this is about 3640 kcal. This corresponds with a weight gain of about 0.5 kg during a treatment period. Subjects will be informed on this fact and advised on how to reduce energy intake from their normal diet.

The oral methionine loading test as performed in the present study (100 mg/kg body weight) is routinely used in hospitals to diagnose hyperhomocysteinemia. It was also applied in two intervention studies previously carried out at TNO in which healthy subjects daily ingested a placebo treatment or betaine (dosages 1.5g/day to 6 g/day) for 6 weeks. Adverse reactions were reported after (repeated) methionine loading, including faintness, nausea, abdominal complaints, headache, tiredness and blurred vision. Cottington et al. (28) reported the death of a control subject after an oral load of methionine for a study of the possible relationship between homocysteine and Alzheimer's disease. The most likely explanation for this adverse event was a tenfold overdosage of methionine. A number of studies have demonstrated that endothelial function is impaired temporarily following methionine loading (29-31). Recently, the safety and adverse effects of the methionine-loading test were evaluated (32). Acute complications during a standard methionine loading test (100 mg/kg body weight) and the 30 d mortality were assessed in 296 patients with coronary artery or peripheral arterial disease and 591 controls. Acute complications were observed in 33% of the women and 16.5% of the men. Most common symptom was diziness. None of the subjects died. It was concluded that although standard loading with L-methionine frequently causes transitory complications impairing perception and vigilance, the test does not have serious adverse effects on vasculature and may be considered a safe procedure (32).

8.5 Standardized diet

On Days 01, 15, 29 and 43 standard breakfast low in methionine will be provided as described in P4963 F07. Subjects can select the composition of breakfast on Day 01 and have to consume the same breakfast on Days 15, 29, 43. One cup of coffee is allowed with breakfast, but consumption has to be the same on Days 01, 15, 29, 43. Supply of methionine dissolved in a glass of orange juice (ca. 150 ml) will be registered on P4963 F08. During the morning concumption of water is allowed, but consumption of coffee will be restricted to one cup. Lunch will be served without restrictions after collection of the 6h blood sample.

8.6 Compliance with study instructions and treatment

Compliance with consumption of study substances will be checked as described in section 8.2.3. Compliance with dietary restrictions will be checked on form P4963 F06a (pre-study) + F06b (in study).

9 Subjects

9.1 Population base

The subjects in this study will be recruited from the pool of volunteers of TNO Nutrition and Food Research and, if needed, by advertisements in local newspapers.

9.2 Number of subjects and rationale for the number

A total of 26 male subjects will participate in the study

Power calculations have been based on a previous study in which the effects of betaine on plasma homocysteine have been measured. Choline from the diet is absorbed from the intestinal lumen and oxidized to betaine in body tissues. No data are available on the bioavailabilty of choline and the amount of choline that is metabolized into betaine. It is assumed that dietary choline is half as effective as dietary betaine in lowering plasma homocysteine concentrations. Therefore the effect of consumption 1.5 g betaine during 2 weeks on homocysteine concentration (study 3818) was used for the power calculation.

Fasting plasma homocysteine
The standard deviation of the response over the intervention period in the betaine group was 2 µmol. The decrease in plasma homocysteine in the betaine group (1.5 g/d of betaine) relative to placebo was 1.3 µmol/L. With 26 subjects we are able to detect a statistically significant decrease in fasting homocysteine concentration of 1.3 µmol/L with a power of 90% (alpha=0.05, 2-sided test) and a decrease of 1.1 µmol/L with a power of 80% (alpha=0.05, 2-sided test).

Plasma homocysteine following methionine loading
The standard deviation of the response (concentration of homocysteine at t=6 h after methionine loading minus the concentration at t=0 h) over the intervention period in the betaine group (1.5 g/d of betaine) was 6.3 µmol/L. The decrease in homocysteine concentration at t=6 h after methionine loading in the betaine group relative to placebo was 6.3 µmol/L. With 26 subjects we are able to detect a statistically significant decrease in post-methionine loading homocysteine concentration of 4.2 µmol/L with a power of 90% (alpha=0.05, 2-sided test). This is similar to the decrease that was observed after a single dose of 0.75 g betaine in study 3818. With a power of 80% (alpha=0.05, 2-sided test) a decrease of 3.6 µmol/L can be detected.

9.3 Replacement

Subjects will be replaced upon withdrawal before Day 01 of the study.

9.4 Inlusion criteria

1. Healthy males as assessed by the health and lifestyle questionnaire, physical examination and results of the pre-study laboratory tests
2. Age at day 01 of the study: 50-80 years
3. Body Mass Index (BMI) ≤ 33 kg/m^2
4. Normal Dutch eating habits, including use of breakfast

5. Willing not to use supplements containing B-vitamins, lecithin, choline (derivatives) and betaine from the oral information session until the end of the study
6. Voluntary participation
7. Having given their written informed consent
8. Willing to comply with the study procedures, including dietary restrictions
9. Willing to accept use of all nameless data, including publication, and the confidential use and storage of all data
10. Willing to accept the disclosure of the financial benefit of participation in the study to the authorities concerned

9.5 Exclusion criteria

Subjects with one or more of the following characteristics will be excluded from participation:

1. Participation in any clinical trial including blood sampling and/or administration of products up to 90 days before Day 01 of this study
2. Participation in any non-invasive clinical trial up to 30 days before Day 01 of this study
3. Having a history of medical or surgical events that may significantly affect the study outcome, including cardiovascular disease or hypertension
4. Use of medication known to interfere with homocysteine metabolism
5. Plasma total homocysteine concentrations > 26 µmol/L
6. Plasma vitamin B6 concentrations ≤ 15 nmol/L
7. Serum vitamin B12 concentrations < 138 pmol/L
8. Serum folic acid concentrations < 5.0 nmol/L
9. Alcohol consumption > 28 units/week
10. Reported unexplained weight loss or gain of > 2 kg in the month prior to the pre-study screening
11. Reported slimming or medically prescribed diet
12. Reported vegan or macrobiotic
13. Reported food allergy
14. Use of B-vitamin supplements, lecithin, or supplements containing choline (derivatives) or betaine, more than once weekly < 1 month before screening
15. Recent blood or plasma donation (< 1 month prior to the start of the study)
16. Not willing to stop blood or plasma donation during the study
17. Personnel of TNO Nutrition and Food Research, their partner and their relatives in the first and second remove
18. Not having a general practitioner
19. Not willing to accept information-transfer concerning participation in the study, or information regarding his/her health, like laboratory results, findings at anamnesis or physical examination and eventual adverse events to and from his general practitioner

10 Informing the subjects

The volunteers will be informed verbally on the aim, the study procedures, the constraints and insurance of the study and receive a copy of the written information (P4963 B01). Those who wish to participate, will subsequently sign 2 copies of the informed consent form (P4963 F01) and keep 1 copy.

11 Study parameters

11.1 Pre-study screening

11.1.1 Medical History

Medical history will be assessed by an interview by the (assistant) medical investigator on the basis of the filled-in health and lifestyle questionnaire (P4963 F04).

11.1.2 Physical examination

Physical examination will be carried out according to SBJ/111 and written instructions by the (assistant) medical investigator. In addition venous access will be checked. Systolic and diastolic blood pressure and heart rate will be measured (SBJ/102).

Body weight will be measured according to SBJ/101, i.e. with the subject wearing indoor clothing, without shoes, wallet and keys. Height will be measured according to SBJ/104, i.e. without shoes.

11.1.3 Clinical laboratory test

Clinical laboratory tests will be executed in fasting samples and will include:

- Haematology (RBC, WBC, platelets, Ht, Hb, differential white blood cell count)
- Serum clinical chemical profile (gamma-GT, ALAT, ASAT, ALP, glucose, total bilirubin, albumin, creatinine, urea, sodium, potassium, chloride)
- Dipstick urinalysis (protein, glucose, leucocytes, erythrocytes, nitrite, pH, ketones, bilirubin, urobilinogen). If the dipstick test gives values above the normal range for leucocytes, blood or protein, a microscopic inspection of sediment of urine will be done.

The methods used and the applicable normal values are given in P4963 B03 and P4963 B04, respectively.
Results will be reported to the medical investigator for evaluation. A copy of the laboratory report will be sent to the principal investigator.
In case of clinically relevant abnormal laboratory results, the medical investigator can decide to exclude the subject and will inform the subject and the subjects general practitioner.

11.1.4 Total homocysteine and B-vitamins

Plasma total homocysteine, plasma vitamin B6, serum vitamin B12 and serum folic acid concentrations will be determined in fasting blood samples.
The methods used are given in P4963 B03.

11.2 In-study

The in-study parameters are mentioned below and the methods for blood analyses used are summarized in P4963 B03. If analysis is not according to a SOP, a working procedure, approved by the principal investigator and the responsible co-investigator, will be used and the method will be described in the report.

Blood analysis

Total homocysteine will be measured in fasting plasma samples at days 01, 13, 15, 29, 41, 43 and in non-fasting plasma samples collected at 6h after methionine loading on day 01, 15, 29 and 43

Total cholesterol, HDL cholesterol, LDL-cholesterol (calculated), triacylglycerols will be measured in fasting serum samples on days 13, 15, 41 and 43

Gamma-GT, ALAT, ASAT, ALP, creatinine will be measured in fasting serum samples on days 13, 15, 41 and 43

Plasma vitamin B6 and serum vitamin B12 and folic acid concentrations will be measured in fasting blood samples on days 13, 15, 41 and 43

Betaine, dimethylglycine and choline will be measured in fasting and non-fasting plasma samples collected at 6h after methionine loading at day 01, 15, 29, 43.

Blood lipids and liver enzymes will be analysed after each treatment period. The remaining blood parameters will be analysed after completion of the study.

Body weight

Body weight will be measured with the subject wearing indoor clothing without shoes, wallet and keys on day 01, 15, 29, 43

11.2.1 Additional parameters

On all blood sampling occasions, plasma aliquots are stored for possible later analysis of methionine (and other amino acids), S-adenosylmethionine (SAM), S-adenosylhomocysteine (SAH), choline and betaine metabolites, and cholesterol derivatives. Additional on day 13 blood will be stored for possible later analysis of polymorphisms associated with homocysteine metabolism, folate metabolism and/or choline metabolism.

11.2.2 Adverse events

A well-being questionnaire will be filled-in by the subjects every visit.
Adverse events will be established as described in §12.6.

12 Study procedures

12.1 General

No examinations will be done before the subject concerned gave her/his written informed consent.

For both the pre-study period and the treatment period checklists per subject will be used (P4963 forms F02 and F03, respectively).

12.2 Pre-study screen, eligibility and selection

Subjects having informed TNO of being interested in possible study participation will be invited to come to TNO for an oral information session on the study.

At the start of the oral information session, the "written information for subjects" (P4963 B01, in Dutch) will be handed out. After oral information a booklet will be handed out containing the following forms:
1. Form P4963 F02 (subject checklist (pre-study))
2. Form P4963 F01 (written informed consent; in duplicate, in Dutch)
3. Form P4963 F04 (health and lifestyle questionnaire, in Dutch)
4. Form: Physical examination
5. Form: Eligibility checklist
6. Form: Participation history
7. Form: End of trial

Subjects willing to participate will be asked, at first, to undersign both informed consent forms, thereafter to complete the health and lifestyle questionnaire and the food questionnaire.
Subjects will receive one of the signed (subject and TNO) informed consent forms.
Each subject will be allocated to a pre-entry number consisting of the TNO study code (4963), followed by a slash ('/'), followed by a 3-digit number starting at 101.

The medical investigator will inform in writing the general practitioner of each subject who has signed the informed consent form on his/her application for study participation.

The subjects will have a pre-study screening before the start of the treatment period. The pre-study screening will involve:
1. An interview (anamnesis) with the medical investigator on basis of the completed health and lifestyle questionnaire
2. Urine and blood collection (fasting) for clinical laboratory tests
3. Blood collection (fasting) for analysis of homocysteine and vitamin B6, B12 and folic acid.
4. Physical examination

Based on the results of the pre-study screening, the medical investigator will establish the eligibility. From the eligible subjects, males with the highest homocysteine concentrations (though not exceeding 26 µmol/L) will be selected. For this purpose we aim at having approximately 40 eligible subjects to select 26. The principal investigator

will inform in writing subjects when they are eligible and whether they will be invited to participate in the study or are reserve or whether they are not selected. The medical investigator will inform the subjects when they are not eligible.

12.3 Allocation to entry number and study treatment

After inclusion of all subjects, the statistician will randomly allocate the subjects to an entry number and a treatment order, with randomization restricted by plasma homocysteine concentration and smoking.
Entry numbers will consist of the TNO study code (4963), followed by a slash ('/'), followed by a 2-digit number (01-26).

The statistician will inform in writing the Senior Research Nurse and dietician on allocation to entry number and the dietitian on allocation of entry number to study treatment (see also section 8.2.2).

12.4 Assessments

The assessments to be done are summarized in the scheme given in §3.

For logistic reasons, subjects will be divided in two groups, starting their treatment period on two consecutive days. For both series the first day of treatment will be specified as Day 01. All subsequent visits will be divided in these two groups, with visits on two consecutive days.

During the whole treatment period, subjects are not allowed to consume liver, spinach and shrimps; consumption of liver products will be restricted to 2x per week. Also consumption of eggs or egg-containing products will be prohibited or restricted and consumption of products containing lecithin will be restricted. Dietary restrictions start two weeks before day 01 of the study and are described in P4963 B01.

On **Days 01 and 29** subjects will visit TNO at ca. 08:00 h in a fasting state (from 22:00 h the evening before). After filling in a well-being questionnaire, body weight will be measured. Fasting blood samples will be collected and subsequently subjects will ingest a methionine dose (100 mg L-methionine per kg body weight; t=0 h) dissolved in a glass of orange juice (P4963 F08) together with a standard breakfast (P4963 F07) and one portion of study substance mixed with about 200 mL of custard. Blood samples will be collected at 6 h following ingestion of the methionine dose (t=6 h). A lunch without restrictions will be served afterwards. Subjects reveive study substances and a diary for the period until the next visit and will be instructed about the storage conditions of the study substances and about how to consume the substances.

On **Days 05 (starting group 1) or 06 (starting group 2) and 35** subjects will visit TNO to fill-in a well-being questionnaire, to check compliance and to collect study substances for the remaining of the treatment period.

On **Days 13 and 41**, subjects will visit TNO between ca. 08:00 and ca. 09:30 h in a fasting state (from 22:00 h the evening before). Subjects have to fill-in a well-being questionnaire. After collection of fasting blood samples, breakfast will be served together with one portion of study substance.

On **Days 15 and 43**, subjects will visit TNO at ca. 08:00 h in a fasting state (from 22:00 h the evening before). Compliance will be checked. After filling in a well-being questionnaire, body weight will be measured. Fasting blood samples will be collected and subsequently subjects will ingest a methionine dose (100 mg L- methionine per kg body weight) dissolved in orange juice (t=0 h) together with a standard breakfast and one portion of study substance mixed with 200 mL of custard. Blood samples will be collected at 6 h following ingestion of the methionine dose (t=6 h). A lunch without restrictions will be served afterwards.

12.5 Compliance

During the treatment periods, subjects will be asked to register the intake of study substances on form P4963 F06b. On this form they will also report deviations from the dietary restrictions. Subjects will have to return unused study substances. The number of study substances returned, registered on form P4963 F03, will be used to check compliance. In addition the empty bowls will be returned and weighed to assess the remaining of the study substances. Compliance to dietary restrictions during the pre-study period and the wash-out period will be checked with form P4963 F06a.

12.6 Adverse Events (AEs)

AEs will be established by the medical investigator on basis of:
1. Answer to the open question: 'How are you feeling?
2. Spontaneous reporting
3. Well-being questionnaire (Form P4963 F05)

AEs will be classified under the responsibility of the medical investigator according to ICD-10 (published by the WHO) and (P4963 B05). He/she will register his/her findings, conclusions and actions. Increased homocysteine concentrations in plasma following the methionine loading test will not be considered as an adverse event. Additional tests on medical indication can be performed, if required as assessed by the medical investigator.

12.7 Serious Adverse Events (SAEs)

Any serious adverse event, whether or not related to the study treatment will be reported by the medical investigator immediately (within 24 hours) to the principal investigator, the sponsor or its representatives, the TNO Medical Ethics Committee (TNO-METC), the TNO-management, the general practitioner and the study subject. The exception to this rule is that serious adverse events that have already been treated by the general practitioner or medical specialist, or about which the general practitioner or medical specialist have already been informed, which situation is to be judged by the medical investigator, do not have to be reported to the general practitioner or subject. A written report on the event will be sent to the sponsor and the TNO-METC within three working days.
See P4963 B05 for a definition of a serious adverse event.

12.8 Safety

The medical investigator will guard the medical safety of the subjects within the frame work of the study. He/she is on call 24 hours per day. During visits with blood collection blood will be collected by an authorized person (authorized by the Medical

Investigator), and a registered nurse or the (assistant) medical investigator will be present in the research facility.

12.9 Post-study screen

At the last day of the study (or within one week after the clinical part) subjects will have a post-study screen. The post-study screen will involve an interview with the medical investigator.

12.10 Criteria for withdrawal or premature discontinuation

Subjects may discontinue the trial at any moment without the obligation to state the reason for discontinuation.
Subjects may be withdrawn from the study by the principal investigator if they do not comply with the rules and regulations of the study.
Subjects may be withdrawn from the study by the medical investigator in case of reported serious adverse events or in case of other medical/social/psychological events as evaluated by the medical investigator and discussed with the principal investigator.
Each subject who does not complete the study for any reason should de contacted ay least at the end of the particular study day and should have a post-study screen, if requested by the subject or medical investigator.

13 Code and handling of samples

13.1 Sample coding

Blood samples and spot-urine samples collected at the **pre-study** screen will be coded as follows:
study code (4963), followed by a slash ('/'), followed by the pre-entry number (3 digits), followed by a slash ('/'), followed by the visit-time code (VTC, P4963 B02), followed by (next line) tube code for the type of matrix and volume.

In study blood samples collected will be coded as follows: Study code (4963), followed by a slash ('/'), followed by the entry number (2 digits), followed by a slash ('/'), followed by the visit time code (VTC, P4963 B02), followed by (next line) tube code for type of matrix and volume.
For sub-sample tubes, on the second line the tubecode number, type of sample and type of analyses will be indicated. In case more than one sample per type is present, this will be followed by a one-digit number

13.2 Sample collection, preparation and storage

13.2.1 Sample collection pre-study

A total of about 22 mL blood will be collected for screening.

For haematology, blood will be collected in Vacutainer® tubes (2 ml) containing K_3EDTA as anticoagulant, coded '02-EDTAbl', stored at room temperature and analysed on the day of collection. Non used material will be discarded after analysis.

For serum collection, blood will be collected in Vacutainer® tubes (9.5 ml) containing clot activator and a gel to separate serum and packed cells after centrifugation, coded '05-stolbl'. The samples will be centrifuged 15-30 min after collection, for 15 min at ca. 2.000 × g at ca. 4°C. After centrifugation serum will be removed and mixed and divided as follows:
- A 2.0 mL aliquot will be used for serum chemistry (05-ser.hit) and stored at ≤ -18°C
- A 1.5 mL aliquot will be used for analyses of vitamin B12 and folic acid (05-ser.B/F) and stored at ≤ -18°C
- Remaining serum (aliquot of max. 2 mL will be stored as archive serum at ≤ -70°C (05-ser.arc)

For plasma collection, blood will be collected in Vacutainer® tubes (10 ml) containing EDTA, coded 12-EDTAbl. Immediately after collection blood will be mixed well and put on ice. Samples will be centrifuged within 15-30 min after collection for 15 min at ca. 2.000 × g and ca. 4°C. After centrifugation plasma will be removed and divided as follows:
- A 1.0 mL aliquot will be used for analyses of homocysteine (12-pl.homcy)
- A 1.5 mL aliquot will be used for analyses of vitamin B6 (12-pl.vitB6)

Remaining plasma (aliquots of max. 2 mL) will be stored as archive (12-pl.arc). All plasma samples will be stored at ≤ -18°C.

Spot urine samples, coded ’04-uri.spot', will be stored at room temperature and analysed on the day of collection (dipstick) and discarded after analysis.
A microscopic inspection of sediment of urine will be done if the dipstick test gives values above the normal range for leucocytes, blood or protein.

Pre-study blood and urine sample collection, subsample presence and transfer will be registered on forms P4963 F09 and P4963 F10 .

13.2.2 Sample collection in-study

For serum collection, blood will be collected in Vacutainer® tubes (9.5 ml) containing clot activator and a gel to separate serum and packed cells after centrifugation, coded ’05-stolbl'. The samples will be centrifuged 15-30 min after collection, for 15 min at ca. 2.000 × g at ca. 4^oC. After centrifugation serum will be removed and mixed and divided as indicated below. All serum samples, except those meant for analysis of blood lipids and liver enzymes, will be stored at $\leq$ - 70^oC. Samples for analysis of blood lipids and liver enzymes will be stored at $\leq$ -18^oC.

For plasma collection, blood will be collected in Vacutainer® tubes (10 ml) containing EDTA, coded 12-EDTAbl. Immediately after collection blood will be mixed well and put on ice. Samples will be centrifuged within 15-30 min after collection for 15 min at ca. 2.000 × g and ca. 4^oC. After centrifugation plasma will be removed and divided as indicated below. Archive plasma samples will be stored at $\leq$ -70^oC, all other plasma samples will be stored at $\leq$ -18^oC.

For heparin plasma collection, blood will be collected in Vacutainer® tubes (5 ml) containing Lithium-heparin as anti-coagulant, coded ‘03-li.hep.bl'. Immediately after collection, blood will be mixed well and put on ice. Within 30 min after collection samples will be centrifuged for 15 min at ca. 2.000 × g at ca. 4^oC. After centrifugation plasma will be removed and divided over the aliquots indicated below. All samples will be stored at $\leq$ -70^oC.

<u>Days 01 and 29 (total about 30 ml blood)</u>
One tube of 10 mL EDTA blood (fasting) will be collected at t=0 and plasma will be divided as follows:
1 mL for homocysteine
1 mL for repeat analysis
0.5 mL for betaine, dimethylglycine and choline; directly to be put on ice until storage
1.0 mL for pm analysis (SAH/SAM); add 0.625 ml 10% perchloric acid and mix
0.5 mL as archive material

One tube of 5 mL lithium heparine blood (fasting) will be collected at t=0 and plasma will be divided as follows
2 x 0.5 mL for pm analysis of methionine and other amino acids
2 x 0.5 mL as archive material

One tube of 10 mL EDTA blood (post methionine) will be collected at t=6 and plasma will be divided as follows:
1 mL for homocysteine
1 mL for repeat analysis
0.5 mL for betaine, dimethylglycine and choline; directly to be put on ice until storage
1 mL for pm analysis (SAH/SAM); add 0.625 ml 10% perchloric acid and mix
0.5 mL as archive material

One tube of 5 mL lithium heparine blood (post methionine) will be collected at t=6 and plasma will be divided as follows:
2 x 0.5 mL for pm analysis of methionine and other amino acids
2 x 0.5 mL as archive material

<u>Days 15 and 43 (total about 50 mL blood)</u>
Two tubes of 10 mL EDTA blood (fasting) will be collected at t=0 and plasma will be divided as follows:
1 mL for homocysteine
1 mL for repeat analysis
1 mL for vit B6
0.5 mL for betaine, dimethylglycine and choline; directly to be put on ice until storage
1 mL for pm analysis (SAH/SAM); add 0.625 ml 10% perchloric acid and mix
3x 1 mL as archive material

One tube of 9.5 mL clot blood (fasting) will be collected at t=0 and serum will be divided as follows:
2 mL for blood lipids and liver enzymes
1 mL for vitamin B12 and folic acid
1 mL as archive material

One tube of 5 mL lithium heparine blood (fasting) will be collected at t=0 and plasma will be divided as follows:
2 x 0.5 mL for pm analysis of methionine and other amino acids
2 x 0.5 mL as archive material

One tube of 10 mL EDTA blood (post methionine) will be collected at t=6 and plasma will be divided as follows:
1 mL for homocysteine
1 mL for repeat analysis
0.5 mL for betaine, dimethylglycine and choline; directly to be put on ice until storage
1 mL for pm analysis (SAH/SAM); add 0.625 ml 10% perchloric acid and mix
0.5 mL as archive material

One tube of 5 mL lithium heparine blood (post methionine) will be collected at t=6 and plasma will be divided as follows
2 x 0.5 mL for pm analysis of methionine and other amino acids
2 x 0.5 mL as archive material

<u>Days 13 and 41 (total about 30 mL blood)</u>

Two tubes of 10 mL EDTA blood will be collected (fasting) and plasma will be divided as follows:
1 mL for homocysteine
1 mL for vit B6
1 mL for repeat analysis
1 mL for pm analysis
2 x 1 mL as archive material

One tube of 9.5 mL clot blood will be collected (fasting) and serum will be divided as follows:
2 mL for blood lipids and liver enzymes
1 mL for vitamin B12 and folic acid
1 mL as archive material

Additional at day 13 (about 5 mL blood)
For pm blood for polymorphisms whole blood will be collected in Vacutainer® tubes (4.5 mL) containing EDTA coded (15-EDTAbl). After collection the tube should be gently rolled horizontally between the hands and divided gently over three tubes of 0.5 mL. DO NOT SHAKE THE TUBE. Tubes should be stored at $\leq$ -18°C.

During the clinical part about 235 mL blood will be collected. In study blood sample collection, subsample presence and transfer will be registered on forms P4963 F11, P4963 F12, P4963 F14, P4963 F15, P4963 F16, P4963 F17 and P4963 F18.

13.3 Remainder samples and archive samples

Remainder of serum (and plasma) of the screening is meant for repeated analysis in case of unexplained abnormal values and will be stored until subject selection has been completed.

Remainder samples of the study period will be discarded after approval of the final report of the study by the sponsor. Archive samples and non-used samples meant for repeat analysis, pm analysis or additional analysis (see section 11.2.1) of the study period will be discarded 5 years after approval of the final report of the study by the sponsor.

14 Documentation

The documentation of this study consists of the study protocol, correspondence, report, raw data, source documents or authenticated copies of these.

For privacy reasons, documents containing data of individual subjects will be identified only by their pre-entry or entry number.

15 Data management and statistics

15.1 Data management

The following SAS datasets will be prepared:
1. Pre-screening parameters
2. Body weight (days 01, 15, 29, 43)
3. Fasting homocysteine (days 01, 13, 15, 29, 41, 43)
4. Homocysteine after methionine (days 01, 15, 29, 43)
5. Blood lipids and liver enzymes (days 13, 15, 41 and 43)
6. Vitamin B6, B12 and folic acid (days 13, 15, 41 and 43)
7. Betaine, dimethylglycine, choline (days 01, 15, 29 and 43)

For data set 1 raw data will partly be delivered on paper and entered using double data-entry and partly in electronic format. For data set 2 raw data will be delivered on paper and entered using double data-entry. For data set 3, 4, 5, 6 and 7 raw data will be delivered in electronic format /on-line.

Raw data will be registered and will be converted to SAS data. All tranformations and changes in datasets will be controlled and registered. Locked datasets will be electronically transferred to the sponsor.

15.2 Missing values

After completion of the clinical part of the study and before start of datamanagement the principal investigator will decide what has to be done with missing values and will inform datamanagement. Decisions will be described in the report.

15.3 Statistical analysis

15.3.1 Descriptive parameters

Subject characteristics, adverse events and compliance will be presented descriptively.

15.3.2 Derived parameters

For all fasting parameters (homocysteine, blood lipids, liver enzymes, B-vitamins) determined on day 13 and 15 as well as on days 41 and 43 mean values will be used for statistical analysis.

The increase of plasma homocysteine after methionine loading will be calculated at day 01, 15, 29 and 43 by substracting the value at 6h from the value at 0h (pre-loading).

15.3.3 Analysis

ANOVA will be performed using the below given table. If the analysis of variance indicates a treatment effect ($p < 0.05$), comparisons between treatment means of the parameters will be performed using a 2-sided Student t-test.

In case of unequal group sizes, General Linear Models (GLM) procedure will be used in stead of ANOVA.

ANOVA table

Source of variation	Degrees of freedom
Subjects	25
Periods	1
Treatments	1
Residual error	24
Total	51

Based on results and discussion with co-researchers, sponsor and remarks of referees additional statistical analysis may be performed. Additional statistical analysis will be described in an amendment to the protocol.

In all statistical tests performed, the null hypothesis (no treatment effect) will be rejected at the 0.05 level of probability.

16 Reporting

The CRO will draft a report (in English), including a Summary, Introduction, Methods section and Description of results section. The report will include deviations from the protocol and safety data. Results of all parameters as specified in this protocol will be presented in summarizing tables. Individual data will be presented in appendices. The final report will contain an audit certificate by the Quality Assurance Unit (QAU) of the TNO Nutrition and Food Research and a statement on GCP compliance signed by the principal investigator.

17 Publication

Wageningen Centre for Food Sciences (WCFS) is responsible for the writing of a scientific publication concerning the results of the study.

18 Timing

Provided approval of the TNO METC is obtained end March 2003, the planned time schedule will be as follows:

- Start Recruitment and selection	:	March/May 2003
- Study initiation	:	End May 2003
- Completion clinical part	:	Mid July 2003
- Completion lab analysis	:	End August 2003
- Completion statistical analysis	:	Mid October 2003
- Draft unaudited TNO Report	:	Mid November 2003

The actual start and termination dates of the study will be recorded in the final report.

<u>Frequency of communication</u>

The progress of the study will be reported to the sponsor at the following stages, in principle by telephone or e-mail

- Approval of the protocol by the METC
- Start recruitment
- Start screening
- Completion recruitment and selection
- Start clinical part
- End of the first treatment period
- Start of the second treatment period
- End of clinical part
- Start of lab analysis including time schedule
- Deviations from the given time schedule, if any, will be communicated/discussed
- Delivery results of lab analysis; time schedule on statistical analysis and reporting
- Regularly during the reporting phase (every 3 weeks)

Comments from the sponsor on the draft report are expected 4 weeks after delivery

19 Insurance of subjects

Atthe request of Wageningen Centre for Food Sciences TNO has insured the volunteers in this study for damage or death caused by the participation in this study. The insurance company is:

Name	: Winterthur Schadeverzekering Maatschappij
Address	: Postbus 83000<br>1080 AA Amsterdam
Tel.	: 020 5411754
Fax.	: 020 6428428
Policy nr.	: V00615407

The insurance covers damage with a maximum of 453,000.- euro per subject, with a maximum of 6,806,000.- euro for the whole study and is limited to 9,075,605.- euro per insurance year.

Exclusion criteria and insurance conditions will be presented in the written information for volunteers

The Dutch law will prevail in case of legal dispute with a volunteer.

The volunteers will be informed how to handle in case of damage in the written information for volunteers (Appendix P4963 B01).

20 Ethics and quality

20.1 General

The study will be conducted in compliance with the protocol and all amendments to the protocol. The protocol and all amendments to the protocol effecting the design, rationale or objectives of the study, or the burden of or health risks for the volunteers will only be implemented after written approval of the TNO METC (or the CCMO when applicable) and the sponsor. All amendments will be sent to the Medical Ethics Committee for information or approval.

The study , except for the analysis of betaine, dimethylglycine and choline in plasma samples, will be conducted according to:
1. The current revision of the World Medical Association Declaration of Helsinki. 52nd WMA General Assembly, Edinburgh, Scotland, October 2000.
2. The ICH Guidelines for Good Clinical Practice (ICH Topic E6, adopted 01-05-1996 and implemented 17-01-1997)
3. Regulations on Medical Research involving Human Subjects (Medical Research involving Human Subjects Act, Wet medisch-wetenschappelijk onderzoek met mensen, WMO 1999)
4. The current national regulations.

20.2 Good Clinical Practice

TNO Nutrition and Food Research has been inspected by the Public Health Inspectorate for compliance with the principles of good Clinical Practice for the conduct of clinical trials according to the EC directive 91/507/EC and the ICH Guidelines for Good Clinical Practice (CPMC)/ICH/135/95). A written statement is supplied in P4963 B06.

20.3 Quality assurance and monitoring

The Quality Assurance Unit (QAU) of TNO Nutrition and Food Research will conduct audits during te study and will review the final report as required by the ICH Guidelines for Good Clinical Practice (GCP). The audit certificate of the QAU will specify the dates of inspections and reports to management and to the principal investigator.

To assure the quality of the homocysteine analyses an agreement will be made with the responsible laboratory including a description of the methods, materials and apparatus used, the use of control samples and validation of the analytical method. The facilities involved can be inspected by the QAU of TNO Nutrition and Food Research. The analysis of betaine, dimethylglycine and choline carried out in Norway in the lab of Prof. P. Ueland under responsibility of WCFS and will not be audited by TNO Quality Assurance, thus will be excluded from the GCP compliance statement.

Members of the TNO-METC, representatives of the sponsor or regulatory authorities may conduct inspections of the testing facility and/or the raw data and will have direct access to medical records of participants as produced and filed by the TNO Medical Investigator. To prevent interference with the study procedures, appointments for visits are requested in advance.

21 Retention of records, samples and specimens

The following documents will be retained in the archives of the TNO Nutrition and Food Research during 15 years after reporting of the study:
1. Master copies of the approved study protocol and final report
2. All documents containing personal data of individual trial subjects
3. Raw data (source documents or copies of these)
4. Correspondence
5. All other information related to tests and analyses conducted (including records of homocysteine measurements conducted at Wageningen University)

The Wageningen Centre of Food Sciences will be responsible for archiving the raw data on the analysis of betaine, dimethylglycine and choline in plasma samples.

The following samples and specimens will be retained in appropriate facilities of TNO Nutrition and Food Research.

1. A representative part of the test substance will be retained for 5 years if its nature allows it
2. The non-used study samples (samples meant for repeat analysis, pm analysis, additional analysis or archive blood samples) will be retained for 5 years after the final report has been approved by the sponsor (See 13.3).

22 References

1. Ueland, PM, Refsum, H, Beresford, SA, Vollset, SE (2000) The controversy over homocysteine and cardiovascular risk. Am. J. Clin. Nutr. 72: 324-332.
2. van Oort, F.V.A., Melse-Boonstra, A., Brouwer, I.A., Clarke, R., West, C.E., Katan, M.B., Verhoef, P. (2002) Folic acid and plasma homocysteine reduction in older adults: a dose-response study. Submitted.
3. Anonymous (1998) Lowering blood homocysteine with folic acid based supplements: meta-analysis of randomised trials. Homocysteine Lowering Trialists' Collaboration. BMJ 316: 894-898.
4. Smolin, LA, Benevenga, NJ, Berlow, S (1981) The use of betaine for the treatment of homocystinuria. J. Pediatr. 99: 467-472.
5. Wilcken, DE, Wilcken, B, Dudman, NP, Tyrrell, PA (1983) Homocystinuria--the effects of betaine in the treatment of patients not responsive to pyridoxine. N. Engl. J. Med. 309: 448-453.
6. Brouwer, IA, Verhoef, P, Urgert, R (2000) Betaine supplementation and plasma homocysteine in healthy volunteers. Arch. Intern. Med. 160: 2546-2547.
7. Steenge, G.R., Verhoef, P., Katan, M.B. (2002) Effect of betaine on plasma concentrations of fasting and post-methionine loading homocysteine in healthy volunteers. Submitted.
8. Olthof, M.R., van Vliet, T., Boelsma, E., Verhoef, P. (2002) Effect of betaine on plasma homocysteine. A dose-response study in healthy volunteers. In preparation.
9. Zeisel, SH (1981) Dietary choline: biochemistry, physiology, and pharmacology. Annu. Rev. Nutr. 1: 95-121.
10. Food and Nutrition Board, IOM. 2000. Dietary references intakes for thiamin, riboflavin, niacin, bitamin B6, folate, vitamin B12, pantothenic acid, biotin, and choline. Washington DC, National Academy Press.
11. Zeisel, SH, Da Costa, KA, Franklin, PD, Alexander, EA, Lamont, JT, Sheard, NF, Beiser, A (1991) Choline, an essential nutrient for humans. FASEB J. 5: 2093-2098.
12. Buchman, AL, Dubin, M, Jenden, D, Moukarzel, A, Roch, MH, Rice, K, Gornbein, J, Ament, ME, Eckhert, CD (1992) Lecithin increases plasma free choline and decreases hepatic steatosis in long-term total parenteral nutrition patients. Gastroenterology 102: 1363-1370.
13. Buchman, AL, Ament, ME, Sohel, M, Dubin, M, Jenden, DJ, Roch, M, Pownall, H, Farley, W, Awal, M, Ahn, C (2001) Choline deficiency causes reversible hepatic abnormalities in patients receiving parenteral nutrition: proof of a human choline requirement: a placebo-controlled trial. J. Parenter. Enteral. Nutr. 25: 260-268.
14. Zeisel, SH & Blusztajn, JK (1994) Choline and human nutrition. Annu. Rev. Nutr. 14: 269-296.
15. Davis, KL, Berger, PA, Hollister, LE (1975) Letter: Choline for tardive dyskinesia. N. Engl. J. Med. 293: 152
16. Growdon, JH, Hirsch, MJ, Wurtman, RJ, Wiener, W (1977) Oral choline administration to patients with tardive dyskinesia. N. Engl. J. Med. 297: 524-527.
17. Lawrence, CM, Millac, P, Stout, GS, Ward, JW (1980) The use of choline chloride in ataxic disorders. J. Neurol. Neurosurg. Psychiatry 43: 452-454.
18. Zeisel, SH, Wishnok, JS, Blusztajn, JK (1983) Formation of methylamines from ingested choline and lecithin. J. Pharmacol. Exp. Ther. 225: 320-324.

19. Boyd, WD, Graham-White, J, Blackwood, G, Glen, I, McQueen, J (1977) Clinical effects of choline in Alzheimer senile dementia. Lancet 2: 711
20. Cersosimo, RJ & Matthews, SJ (1987) Hepatotoxicity associated with choline magnesium trisalicylate: case report and review of salicylate-induced hepatotoxicity. Drug Intell. Clin. Pharm. 21: 621-625.
21. Chawla, RK, Wolf, DC, Kutner, MH, Bonkovsky, HL (1989) Choline may be an essential nutrient in malnourished patients with cirrhosis. Gastroenterology 97: 1514-1520.
22. Wurtman, RJ, Hirsch, MJ, Growdon, JH (1977) Lecithin consumption raises serum-free-choline levels. Lancet 2: 68-69.
23. Fiume, Z (2001) Final report on the safety assessment of lecithin and hydrogenated lecithin. Int. J. Tox icol. 20: 21-45.
24. Johnson, DW, Mokler, DJ (2001) Lecithin's therapeutic effects. Continuing education module. Massachusetss College of Pharmacy aand Health Sciences. pp 1-7.
25. Yamamoto, K, Asakawa, H, Tokunaga, K, Watanabe, H, Matsuo, N, Tokimitsu, I, Yagi, N (2001) Long-term ingestion of dietary diacylglycerol lowerd serum triacylglycerol in TypeII diabetic patients with hypertrigllyceridemia. J. Nutr. 131: 3204-3207.
26. Nagao, T, Watanabe, H, Goto, N, Onizawa, K, Taguchi, H, Matsuo, N, Yasukawa, H, Itakura, H (2000) Dietary diacylglycerol supresses accumulation of body fat compared to triacylglycerol in men in a double blind controlled trial. J. Nutr. 130: 792-797.
27. Soni, MG, Kimura, H, Burdock GA (2001) Chronic study of diacylglycerol in rats. Food and Chem. Tox. 39: 317-329.
28. Cottington, EM, LaMantia, C, Stabler, SP, Allen, RH, Tangermann, A, Wagner, C, Zeisel, SH, Mudd, SH (2002) Adverse event associated with methionine loading test. A case report. Aterioscler. Thromb. Vasc. Biol. 22: 1046-1050.
29. Bellamy, MF, McDowell, IF, Ramsey, MW, Brownlee, M et al. (1998) Hyperhomocysteinemia after an oral methionine load acutely impairs endothelial function in healthy adults. Circulation 98:1848-1852.
30. Kanani, PM, Sinkey, CA, Browning, RL, Allaman, M, Knapp, HR, Haynes, WG (1999) Role of antioxidant stress in endothelial dysfunction produced by experimental hyperhomocysteinemia in humans. Circulation 100:1161-1168.
31. Hanratty, CG, McGrath, LT, McAuley, DF, Young, IS, Johnstin, GD (2001) The effects of oral methionine and homocysteine on endothelial function. Heart 85:326-330.
32. Krupkova-Meixnerova, l, Vesela, K, Vitova, A, Janosikova, B, Andel, M, Kozich V (2002) Methionine-loading test: evaluation of adverse effects and safety in an epidemiological study. Clinical Nutrition 21: 151-156.

23 Approval of the protocol

23.1 Sponsor

P. Verhoef, PhD

	Signature ____________________	Date (dd-mm-yy) ___-___-___

23.2 TNO Nutrition and Food Research

E.J. Brink, PhD<br>Principal investigator	Signature ____________________	Date (dd-mm-yy) ___-___-___
W.A.A. Klöpping, MD, PhD<br>Medical investigator	Signature ____________________	Date (dd-mm-yy) ___-___-___
C. Kistemaker, BSc<br>Statistician	Signature ____________________	Date (dd-mm-yy) ___-___-___
A.F.M. Kardinaal, PhD<br>TNO Management	Signature ____________________	Date (dd-mm-yy) ___-___-___

24 List of Appendices

P4963 B01 : Written information for subjects (in Dutch)
P4963 B02 : Visit time codes (VTCs)
P4963 B03 : Methods of clinical laboratory tests
P4963 B04 : Reference values of clinical laboratory tests
P4963 B05 : Adverse Events: definitions and codes
P4963 B06 : Statement of GCP compliance
P4963 B07 : Distribution list
P4963 B08 : Composition of study substances

25 List of forms

P4963 F01	:	Written informed consent (in Dutch)
P4963 F02	:	Subjects checklist (pre-study)
P4963 F03	:	Subjects checklist (in study)
P4963 F04	:	Health and lifestyle questionnaire (in Dutch)
P4963 F05	:	Well-being questionnaire (in Dutch)
P4963 F06a	:	Compliance dietary restrictions pre-study/wash-out (in Dutch)
P4963 F06b	:	Daily consumption of the study substances and compliance to ` dietary restrictions in study (in Dutch)
P4963 F07	:	Composition breakfast during methionine loading (in Dutch)
P4963 F08	:	Supply of methionine to the orange juice (in Dutch)
P4963 F09	:	Blood and urine sample collection and transfer (pre-study)
P4963 F10	:	Subsample presence and transfer (pre-study)
P4963 F11	:	Blood sample collection days 01, 29 (in-study)
P4963 F12	:	Blood sample collection days 15, 43 (in-study)
P4963 F13	:	Registration weight methionine portions per subject (in Dutch)
P4963 F14	:	Blood sample collection and transfer day 13 (in-study)
P4963 F15	:	Blood sample collection days 41 (in-study)
P4963 F16	:	Subsample presence, storage and transfer days 01, 29 in study
P4963 F17	:	Subsample presence, storage and transfer days 13, 41 in study
P4963 F18	:	Subsample presence, storage and transfer days 15, 43 in study
