# Supplementary material for: Effect of Homocysteine-Lowering Nutrients on Blood Lipids: Results from Four Randomised, Placebo-Controlled Studies in Healthy Humans
Source: PLoS Med. 2005 May 31;2(5):e135. doi: 10.1371/journal.pmed.0020135 (PMC1140947; doi:10.1371/journal.pmed.0020135)
Supplement: Protocol S3 — (585 KB PDF). [file pmed.0020135.sd003.pdf]

TNO Nutrition and Food Research

Nederlandse Organisatie voor
toegepast-natuurwetenschappelijk
onderzoek / Netherlands Organisation
for Applied Scientific Research

Nutritional Physiology
Location Zeist
Utrechtseweg 48
P.O. Box 360
3700 AJ Zeist
The Netherlands

www.tno.nl

P +31 30 694 41 44
F +31 30 695 72 24
infofood@voeding.tno.nl

TNO Protocol Amendment 01

P4963 | Amendment 01 / Final |

Effect of choline on post-methionine loading and fasting concentrations of plasma homocysteine in healthy volunteers

Date	21th May, 2003
At the request of	Wageningen Centre for Food Sciences
Drafted by	Dr ir E.J. Brink
TNO project number	010.21110
TNO study code	4963
Status	Final
Previous versions	
Number of pages	15
Number of appendices	7

All rights reserved.
No part of this publication may be reproduced and/or published by print, photoprint, microfilm or any other means without the previous written consent of TNO.

In case this report was drafted on instructions, the rights and obligations of contracting parties are subject to either the Standard Conditions for Research Instructions given to TNO, or the relevant agreement concluded between the contracting parties. Submitting the report for inspection to parties who have a direct interest is permitted.

© 2003TNO

Contents

	Page
Reason for the amendment	3
Revised text	5
2. Synopsis	5
3. Schedule of assessments	6
4. Responsible personnel and test facilities	7
6. Objective of the study	8
8. Study substances and study treatments	8
12. Study procedures	11
15. Datamanagement and statistics	13
23. Approval of protocol amendment 01	14
24. List of appendices and forms	15

Reason for the amendment

This amendment describes changes and additions to the revised final protocol dd 19 March 2003 which occurred during the preparatory phase of this study

The proposed reference substance was a commercially available cooking oil consisting mainly of diacylglycerol (ENOVA™). At present this oil is in the novel foods procedure in The Netherlands. Although the product is commercially available in the United States and Japan and the advice of the Health Council of the Netherlands to the Minister indicates that the oil is safe for human consumption, it was not possible to obtain this product from either Japan or the United States for the use in our study. Therefore, Unilever Research Laboratorium in Vlaardingen prepared a reference oil having a fatty acid profile comparable with that of the test substance. As a consequence in the protocol the following sections were changed: § 2, § 8.1.1, § 8.1.2, § 8.2.4, § 8.4, P4963 B08. Since it was known before the information session that it would not be able to obtain this oil, the written information for volunteers (P4963 B01) and the informed consent form (P4963 F01) were adapted accordingly. At that moment it was only clear that we would choose for edible control oil with comparable fatty acid composition. It was decided to describe this in an amendment after the composition of the product was known which turned out to be just before the start of the clinical part.

Since there is no information on the effect of choline intake on plasma homocysteine in healthy adults, it is important to choose the highest achievable dose. The daily dose of choline was set at about 2.5 g divided over two portions per day. Following this approach the effect of a single intake would be measured after consumption of only about 1.25 g choline. Since the present study is the first efficacy study the best approach to answer this question would be to study the acute effect after the highest achievable dose. Due to the strong taste of Phoschol this appeared to be 1.5 g. As a consequence the description of the objectives was adapted in the following sections: § 2 and § 6 and the text of § 8.2.1 was adapted.

It was indicated in sections 2, 4.2, 4.3 and 8.1.4 that analysis of choline in study substances occurred under responsibility of WCFS. Due to practical reasons these analysis were carried out at TNO Nutrition and Food Research. This was adapted in the sections mentioned above.

Analysis of choline in the test product resulted in a mean concentration of choline in the test product of 75 g/kg. This means that to consume 2.5 g choline per day 34 g of Phoschol instead of 30 g per day will be consumed. This is adapted in sections 2, 8.2.1 and 8.4.

The microbiological safety of the test substance when mixed with the custard were tested. Analysis are carried out under responsibility of G. den Reijer. Results are indicated in P4963 B08-revised. The text in sections 4.2 and 4.3 was adapted accordingly.

As indicated in section 8.2.1 the reference oil would be flavoured in order to guarantee double blinding. The amount and type of flavouring is described in this amendment as adaption of section 8.2.1.

Methionine will not be packed in small brown bottles but in beakers. This is adapted in sections 8.2.1 and 8.2.4.

H. Viersen will leave TNO at 01 June 2003. Y. van der Wel took responsibility as deputy Medical Investigator from 01 May 2003. This is adapted in sections 4.2 and 4.3.

The amount of reference substance given will be based on the amount of (energy from) fat in the test substance. This isoenergetic exchange results in an amount of reference substance of 25.5 g per day. This is adapted in sections 2 and 8.2.1 .

There appeared to be a difference between day numbers 05, 06 and 35 as mentioned in the protocol and actual calender data of the scheduled visits. The correct day numbers are 06, 07 and 36. This is now correctly adapted in sections 3, 8.2.3, 12.4, P4963 F03-revised.

With respect to food restrictions it became clear the beets contain a significant amount of betaine, which is a metabolic product from choline and thus could interfere with the study result. It was therefore added to the list of prohibited foods. Selected subjects were informed on this fact in the invitation letter for this study and were asked to write it on the restriction list they received (P4963 B01-revised, bijlage 7). P4963 B01 revised was not adapted any more. In checking compliance to dietary restrictions it was added tot P4963 F06, question 6. The text in section 12.4 is adapted.

Four reserve subjects were allocated to entry number and to treatment order. This is now indicated in section 12.3.

In statistical analyses of the methionine response the effect after single ingestion (short term) of choline and chronic ingestion (long term) of choline will also be taken into account. As a consequemce the ANOVA table was adapted (section 15.3.3)

Forms and appendices

Revised forms and appendices are added to the amendment (P4963 F03-revised, F06, F07-revised, F13-revised). Main reason for changing forms are new ideas for a better evaluation of the conduct of the study. New forms were developed for preparing the mixtures of study substances with custard (P4963 F19, F20 and F21). Since information on these forms will result in deblinding they are not presented in this amendment.

P4963 F01 and P4963 B01 were revised due to the choice of another reference substance. P4963 B08 was revised due to additional information on the study substances and the choice of another reference substance.

Revised text

Only changed or added text is presented in this amendment. This is printed bold and underlined. The presented text replaces the text as given in the protocol. Text which is not applicable anymore is indicated by a single strikethrough.

2. Synopsis

Objectives

- To investigate the effect of daily intake of about 2.5 g choline on fasting plasma homocysteine
- To investigate the effect of daily intake of about 2.5 g choline on post-methionine loading plasma homocysteine
- To investigate the effect of single intake of about **<u>1.5</u>** g choline on post-methionine loading plasma homocysteine

Study substances

- Test substance : Phosphatidylcholine
- Reference substance : **<u>Edible oil mixture with fatty acid profile comparable to the test substance</u>**

Study treatments

- Choline : about 2.5 g/d choline from phosphatidylcholine (this corresponds with **<u>34 g/d</u>** phosphatidylcholine)
- Reference : **<u>25.5 g/d</u>** reference substance (iso-energetic replacement of fat)

Restrictions

- Diet: **<u>No consumption of beets</u>**

Responsibilities

<u>- Analysis of fatty acids in study substances:</u> **<u>WCFS</u>**
<u>- Analysis of choline in study substances:</u> **<u>TNO, Dept. of Food Analysis</u>**

3. Schedule of assessments

	Study day*									
Visit	Pre-study		1	2	3	4	5	6	7	8
Activity	Pre study **	-14	01	**06/ 07¥**	13	15	29	**36**	41	43
Anamnesis	+									
Physical examination	+									
Body weight	+		+			+	+			+
Urine sampling	+									
Start dietary restrictions; to be continued throughout the study		+								
Blood sampling (fasting)	+		+		+	+	+		+	+
Blood sampling (non-fasting)			+			+	+			+
Methionine loading test			+			+	+			+
Well-being questionnaire			+	+	+	+	+	+	+	+
Dispense study substances			+	+	+		+	+	+	
Dispense one portion study substance at breakfast			+		+	+	+		+	+
Return empty bowls				+	+	+		+	+	+
Compliance check				+	+	+		+	+	+
End of trial/Post study screen										+

** Maximal 8 weeks before day 01 of the study

The study will have a staggered start in two groups.

* For each staring group Day 01 is defined as the first day of the clinical part of the study

¥ For the first starting group this visit will be on day **07** of the study, for the second starting groups this visit will be on day **06** of the study

4. Responsible personnel and test facilities

4.2 Testing facility and responsible personnel

Responsible persons at TNO:

Department of Nutritional Physiology

Deputy Medical Investigator
Ms. Y. van der Wel, MSc, MD
Phone : 030 694 49 60
Fax : 030 694 49 28
E-mail : Wel@voeding.tno.nl

Department of Food and Food Supplement Analysis

Ms. I Bobeldijk, PhD
Phone : 030 694 42 82
Fax : 030 695 67 42
E-mail : Bobeldijk@voeding.tno.nl

Department of Risk management and Microbiology
Ms. G.D. den Reijer
Phone : 030 694 47 71
E-mail : G.denReijer@voeding.tno.nl

4.3 Responsibilities

The sponsor is responsible for the financial compensation for the conduct of the study and analysis of fatty acid composition ~~and choline content~~ of the study substances. In addition the sponsor is responsible for analysis of betaine, choline and dimethylglycine in plasma samples obtained during the clinical phase. Moreover the sponsor is responsible for the insurance according to the “WMO”. At request of the sponsor TNO insures the volunteers according to the WMO. Insurances for material damage and accidents during the travel to and from TNO and during the stay at TNO are TNO's responsibility.

Ms. Van der Wel will be responsible for the selection of the subjects, medical aspects of the study, documentation, and interpretation and reporting of the AEs and SAEs in case Ms. Klöpping-Ketelaars is not available.

Ms. I. Bobeldijk will be responsible for the analysis of choline in the study substances

Ms. G. den Reijer will be responsible for microbiological analysis of the study substances mixed with custard

6. Objective of the study

The objectives of the present study are

- To investigate the effect of daily intake of about 2.5 g choline on fasting plasma homocysteine
- To investigate the effect of daily intake of about 2.5 g choline on post-methionine loading plasma homocysteine
- To investigate the effect of single intake of about **1.5 g** choline on post-methionine loading plasma homocysteine

8. Study substances and study treatments

8.1 Study substances

8.1.1 Description

- Test substance : Phosphatidylcholine (Phoschol©, American Lecithin Company, Oxford, US)
- Reference : **Edible oil with comparable fatty acid composition**

Methionine loading test : L-methionine (Methionimum apyrogeen, BUFA B.V., Pharmaceutical products)

The reference substance was produced by Unilever Research Laboratorium in Vlaardingen according to Good Manufacturing Practice. The composition of the reference substance is given in P4963 B08-revised.

8.1.2 Supply to TNO

~~Study~~ **The test** substance is a commercially available product and is obtained directly from the supplier including relevant documentation on the composition and safety of the study substances. ~~The composition available at this moment~~ This documentation is given in P4963 B08 -revised and section 8.4. **The reference substance was supplied to TNO on 21 May 2003. Composition of the reference substance is given in in P4963 B08 -revised.** A dietician not involved in the study attributed codes “A” or “B” to the test and reference substance.

8.1.3 Storage conditions

The **test** substance will be stored at room temperature at a dry place. **The reference will be stored in a refrigirator.**

8.1.4 Chemical analyses

The test substance was analysed for ~~choline concentration and~~ fatty acid distribution before the start of the clinical part under responsibility of WCFS. WCFS supplied the Principal Investigator with results of these analysis at least two weeks before the start of the study. **The reference substance will be analysed for fatty acid distribution as soon as possible under responsibility WCFS and the principal investigator will be supplied with this information as soon as possible. Choline content of the test substance was determined according to SOP DAS/LNC/014 before the start of the**

study. Choline content of the reference substance will be analysed as soon as possible according to SOP DAS/LNC/014. Analysis results of the reference substance will be filed in the study dossier and described in the study report.

8.2 Study treatments

8.2.1 Description

Test treatment	:	about 2.5 g/d choline during 2 wk (corresponds with consumption of **34 g/d** test substance during 2 wk)
Reference treatment	:	**25.5 g/d** reference substance during 2 wks

The amount of reference substance was based on iso-energetic replacement of the amount of fat present in the test substance.

Study substances will be consumed divided over the day, two times per day (breakfast and dinner). Half of the dose will be given each time (each dose mixed in about 200 ml custard). **On the days of the methionine loading test the dose give during the morning will be higher to provide 1.5 g choline. As a consequence on these days the dose given during the evening will provide about 1 g choline. On these days the amount of test substance will be 20 g in the morning portion and 14 g in the evening portion of custard and the amount of reference substance will be 15 g in the morning portion and 10.5 g in the evening portion.**

On the last day of each treatment period (day 15 and 29) only one morning portion has to be consumed.

Treatments will be separated by a wash-out period of 2 wk.

Individual portions of custard mixed with the study substances will be prepared by the dietician not further involved in the conduct of the study according to a written instruction that will be authorized by the Principal Investigator. **Preparation of the individual portions will be registered on P4963 F19, F20 and F21. Samples will be kept for possible later analysis.**The individual portions will be labeled as indicated in section 8.2.4.
~~The test substance contains licorice flavoring. To guarantee double-blind conduct of the study the same type of flavoring has to be used for the reference treatment. The exact type of flavour and the amount to be added is under investigation now and will be available before the start of the study. This will be described in an amendment to the protocol.~~ **To guarantee double blinding the reference substance will be flavoured with 5.8 g 'anijsolie' (lot: 02I02FS-168204; Fagron, Capelle a/d IJssel, The Netherlands) per kg oil. The certificate of analysis is given in P4963 B08-revised.**

Microbiological safety of custard mixed with the test substance was determined after storage during eight days in a refrigerator. Results are given in P4963 B08-revised.

Methionine will be packed in ~~small brown bottles~~ **beakers** in individually labelled portions (100 mg/kg body weight) on basis of the pre-study body weight (P4963 F13-**revised**). Label coding is described in section 8.2.4.

8.2.3 Supply to the subjects

On days 01, **06 or 07, 13** 29, **36 and 41** subjects will be provided with a labelled bag containing tightly closed small bowls containing about 200 mL custard mixed with half of the daily dose of the study substance. When the second visit is on day **06**, the number of portions dispensed on day 01 will be **10**; when the second visit is on day **07** the number of portions dispensed on day 01 will be **12**. The number of portions dispensed on day **06 will be 13 or on day 07 will be 11. The number of portions dispensed on days 13 and 41 is three.** The number of portions dispensed on **day 29 will be 14 and on day 36 will be 9**. On days 01, 13, 15, 29, 41 and 43 one portion will be supplied and consumed at TNO. Dispense of the study substances will be registered on form P4963 F03**-revised.** On days **06 or 07, 13,** 15, **36, 41** and 43 the unused study substances (if any) will be returned to TNO. This will be registered on form P4963 F03**-revised** and on form GEN/022 F02. Shelf life is confirmed before the start of the study (**P4963 B08-revised)**

Subjects will receive instructions for consumption of the portions and for storage of the study substances. Daily intake will be registered on form P4963 F06b.

8.2.4 Labelling

Study substances will be labelled according to a study specific instruction as described in SOP DHDV/ALG/711.

Each small bowl with the study substance will be labelled by a preprinted label, as follows:

First line: TNO study number (4963) followed by a slash, followed by the 2-digit entry number, followed by a slash, followed by a day number.
Second line: portion code (“portie 1” or “portie 2”)

e.g. 4963/01/01
portie 1

Furthermore, each small bowl containing study substance will be labelled with the following text:

“TNO Voeding, studie 4963
Vla met **17 g** phosphatidylcholine of **referentie-olie**
Bewaren in de koelkast”

Each **beaker** containing methionine will be labelled with a preprinted label, as follows:
First line : methionine
Second line : TNO study number (4963) followed by a slash, followed by the 2-digit entry number, followed by a slash, followed by a ‘d’ and day number

e.g. methionine
4963/01/d01

For the first dispense to the subjects on day 01 the labels were used as described in the study protocol.

8.4 Safety of treatment

~~Diacylglycerol (DAG) is a component present in edible fats and is GRAS. DAG oils were used in an intervention trial in dosages providing about 8 g DAG during 12 weeks~~

~~(25) or 16 weeks (26) without reported adverse effects. No information is available on human exposure of higher doses of DAG. In a two-year chronic study in rats no toxicologically significant or treatment related effects of diacylglycerol oil consumption at levels up to 5,3% in the diet were observed (27). This corresponds with about 12 g/kg body weight per day for rats. For a man of 70 kg this means a daily intake of 840 g/day. A consumption of 30 g per day as proposed in the present study results in a margin of safety of 28.~~

The safety as described for 30 g phosphatidylcholine/lecithin also holds for 34 g phosphatidylcholine.

Anise oil consists for 84%-93% of trans anethole (European Pharamcopoeia 3rd edition, supplement 2001, pp 446-448). Anethole has a GRAS status as flavouring agent.The acceptable daily intake of trans-anethole is reported to be 0-2 mg/kg body weight (Joint FAO/WHO Expert Committee on Food Additives, 1998). In the present study 5.8 g anise oil is added to one kg of reference substance. Daily consumption of anise oil from the reference product will be about 148 mg. For the subject with the lowest body weight (71.5 kg) this will result in an intake of 2.07 mg/kg body weight per day. All others will have a intake (far) lower than the maximum acceptable daily intake. Anethole intake from the test substance will be higher. Since this is the first efficacy study in which the effect of choline on plasma homocysteine is evaluated and therefore the highest achievable dose of 2.5 g choline per day is chosen. Using lower doses may go together with the risk of finding no effect. This results in a daily anthole intake from the test substance of 510 mg. For the subject with the lowest body weight this results in a daily intake of 7.08 mg/kg body weight and for the subject with the highest body weight of 5.1 mg/kg body weight per day. Although this is above the acceptable daily intake as laid down by the Expert Committee (1998) we do not foresee health risks. Results of toxicity studies in rats indicate that the no effect level in rats is 250 mg/kg body weight (WHO Food Additives, series 14). Rats were fed up to 22 months. The dosage used in our study is more than 35 times lower and the test substance in only given for 2 weeks. In addition the test substance Phoschol© is freely available at the market in the United States. There is no information on the product label of that indicates a maximum intake level of this test substance. Moreover on the product label as well as in an information leaflet of the supplier it is indated that 3 teaspoons doubles the choline concentration in the blood. Three teaspoons equals 15 ml which in turn already would provide 2.25 mg/kg body weight.

12. Study procedures

12.3 Allocation to entry number and study treatment

After inclusion of all subjects, the statistician randomly allocated the subjects to an entry number and a treatment order, with randomization restricted by plasma homocysteine concentration and smoking. Entry numbers consist of the TNO study code (4963), followed by a slash ('/'), followed by a 2-digit number (01-26). **In addition 4 reserve subjects with entry number 27-30 were allocated to an entry number and treatment order. These subjects only will participate when one of the selected candidates will drop-out before or on day 01 of the study. After day 01 no replacement of drop-out will occur.**

12.4 Assessments

The assessments to be done are summarized in the scheme given in §3.

For logistic reasons, subjects will be divided in two groups, starting their treatment period on two consecutive days. For both series the first day of treatment will be specified as Day 01. All subsequent visits will be divided in these two groups, with visits on two consecutive days.

During the whole treatment period, subjects are not allowed to consume liver, spinach, **beets** and shrimps; consumption of liver products will be restricted to 2x per week. Also consumption of eggs or egg-containing products will be prohibited or restricted and consumption of products containing lecithin will be restricted. Dietary restrictions start two weeks before day 01 of the study and are described in P4963 B01**, except for the beets. Subjects are requested to add this restriction to the list of dietary restrictions via the invitation letter.**

On Days 01 and 29 subjects will visit TNO at ca. 08:00 h in a fasting state (from 22:00 h the evening before). After filling in a well-being questionnaire, body weight will be measured. Fasting blood samples will be collected and subsequently subjects will ingest a methionine dose (100 mg L-methionine per kg body weight; t=0 h) dissolved in a glass of orange juice (P4963 F08) together with a standard breakfast (P4963 F07) and one portion of study substance mixed with about 200 mL of custard. Blood samples will be collected at 6 h following ingestion of the methionine dose (t=6 h). A lunch without restrictions will be served afterwards. Subjects receive study substances and a diary for the period until the next visit and will be instructed about the storage conditions of the study substances and about how to consume the substances.

On **Days 06 (starting group 2) or 07 (starting group 1) and 36** subjects will visit TNO to fill-in a well-being questionnaire, to check compliance and to collect study substances ~~for the remaining of the treatment period~~ **until the next visit.**

On Days 13 and 41, subjects will visit TNO between ca. 08:00 and ca. 09:30 h in a fasting state (from 22:00 h the evening before). **Compliance will be checked.** Subjects have to fill-in a well-being questionnaire. After collection of fasting blood samples, breakfast will be served together with one portion of study substance. **Subjects receive study substances and a diary for the period until the next visit and will be instructed about the storage conditions of the study substances and about how to consume the substances.**

On Days 15 and 43, subjects will visit TNO at ca. 08:00 h in a fasting state (from 22:00 h the evening before). Compliance will be checked. After filling in a well-being questionnaire, body weight will be measured. Fasting blood samples will be collected and subsequently subjects will ingest a methionine dose (100 mg L- methionine per kg body weight) dissolved in orange juice (t=0 h) together with a standard breakfast and one portion of study substance mixed with 200 mL of custard. Blood samples will be collected at 6 h following ingestion of the methionine dose (t=6 h). A lunch without restrictions will be served afterwards.

15. Datamanagement and statistics

15.3 Statistical analysis

15.3.3 Analysis

ANOVA will be performed using the below given table. Short term and long term treatment effects will be taken into account. All statistical tests are based on a two-sided 5% significance level. In case of unequal group sizes, General Linear Models (GLM) procedure will be used in stead of ANOVA.

ANOVA table

Source of variation	Degrees of Freedom
Subject stratum	25
Period stratum	1
Subject.Period stratum	
Treatment	1
Residual	24
Subject.Period stratum[1)]	51
Subject.Period.Units stratum	
Term	1
Treatment*Term	1
Residual	50
Total	103

[1)] The subject.period stratum (df = 2n-1) is the total of subject and period strata

Based on results and discussion with co-researchers, sponsor and remarks of referees additional statistical analysis may be performed. Additional analysis will be described in an amendment to the protocol.

23 Approval of protocol amendment 01

23.1 Sponsor

P. Verhoef, PhD
Project Manager ____________________ ___-___-___
Signature Date (dd-mm-yy)

23.2 TNO Nutrition and Food Research

E.J. Brink, PhD ____________________ ___-___-___
Principal investigator Signature Date (dd-mm-yy)

W.A.A. Klöpping, MD, PhD ____________________ ___-___-___
Medical investigator Signature Date (dd-mm-yy)

C. Kistemaker, BSc ____________________ ___-___-___
Statistician Signature Date (dd-mm-yy)

A.F.M. Kardinaal, PhD ____________________ ___-___-___
TNO Management Signature Date (dd-mm-yy)

24& 25 List of appendices and forms

P4963 B01-revised	:	Written information for subjects (in Dutch): 04 April 2003
P4963 B07	:	Distribution list
P4963 B08- revised	:	Composition of study substances: 21 May 2003
P4963 F01 - revised	:	Written informed consent (in Dutch): 04 April 2003
P4963 F03 - revised	:	Subjects checklist (in study): 14 May 2003
P4963 F07 - revised	:	Composition breakfast during methionine loading (in Dutch): 14 May 2003
P4963 F13 - revised	:	Registration weight methionine portions per subject (in Dutch): 14 May 2003
