# Supplementary material for: Effect of Homocysteine-Lowering Nutrients on Blood Lipids: Results from Four Randomised, Placebo-Controlled Studies in Healthy Humans
Source: PLoS Med. 2005 May 31;2(5):e135. doi: 10.1371/journal.pmed.0020135 (PMC1140947; doi:10.1371/journal.pmed.0020135)
Supplement: Protocol S4 — (470 KB PDF). [file pmed.0020135.sd004.pdf]

TNO Nutrition and Food Research

Nederlandse Organisatie voor toegepast-natuurwetenschappelijk onderzoek / Netherlands Organisation for Applied Scientific Research

Nutritional Physiology
Location Zeist
Utrechtseweg 48
P.O. Box 360
3700 AJ Zeist
The Netherlands

www.tno.nl

P +31 30 694 41 44
F +31 30 695 72 24
infofood@voeding.tno.nl

TNO Protocol Amendment 02

P4963 | Amendment 02 / Final |

Effect of choline on post-methionine loading and fasting concentrations of plasma homocysteine in healthy volunteers

Date	8th September, 2003
At the request of	Wageningen Centre for Food Sciences
Drafted by	Dr ir E.J. Brink
TNO project number	010.21110
TNO study code	4963
Status	Final
Previous versions	--
Number of pages	8
Number of appendices	1

All rights reserved.
No part of this publication may be reproduced and/or published by print, photoprint, microfilm or any other means without the previous written consent of TNO.

In case this report was drafted on instructions, the rights and obligations of contracting parties are subject to either the Standard Conditions for Research Instructions given to TNO, or the relevant agreement concluded between the contracting parties. Submitting the report for inspection to parties who have a direct interest is permitted.

© 2003 TNO

Contents

	Page
Reason for the amendment	3
Revised text	3
2. Synopsis	4
11. Study parameters	5
15. Data management and statistics	6
23. Approval of protocol amendment 02	7
24. List of appendices	8

Reason for the amendment

This amendment describes a change of the revised final protocol dd 19 March 2003 with respect to the study parameters.

Fasting and post-methionine loading homocysteine concentrations in plasma are the main outcome parameters of this study. Betaine, dimethylcholine and choline concentrations in plasma were planned to be measured to elucidate a possible mechanism. At this stage it is decided by the sponsor not to measure betaine, dimethylglycine and choline concentrations in plasma, but first await and report the results of the effect of choline on homocysteine concentrations. The sponsor will decide in a later stage whether or not betaine, dimethylglycine and choline (as well as for the other additional parameters as mentioned in section 11.2.1 of the protocol) will be measured. This decision will be documented in an amendment and filed in the study dossier.

Revised text

Only changed text is presented in this amendment. The presented text replaces the text as given in the protocol. Text which is not applicable anymore is indicated by a single strikethrough.

2 Synopsis

Test parameters in study

Blood analysis

- Total homocysteine will be measured in fasting plasma samples on days 01, 13, 15, 29, 41, 43 and in non-fasting plasma samples collected at 6h after methionine loading on day 01, 15, 29 and 43
- Total cholesterol, HDL cholesterol, LDL-cholesterol (calculated), triacylglycerols in fasting serum samples on day 13, 15, 41 and 43
- gamma-GT, ALAT, ASAT, ALP, creatinine in fasting serum samples on days 13, 15, 41 and 43
- Plasma vitamin B6, and serum vitamin B12 and folic acid in fasting samples on days 13, 15, 41 and 43
- ~~Betaine, dimethylglycine and choline in fasting and non-fasting plasma samples collected at 6h after methionine loading on day 01, 15, 29, 43~~

11 Study parameters

11.2 In-study

Blood analysis

Total homocysteine will be measured in fasting plasma samples at days 01, 13, 15, 29, 41, 43 and in non-fasting plasma samples collected at 6h after methionine loading on day 01, 15, 29 and 43

Total cholesterol, HDL cholesterol, LDL-cholesterol (calculated), triacylglycerols will be measured in fasting serum samples on days 13, 15, 41 and 43

Gamma-GT, ALAT, ASAT, ALP, creatinine will be measured in fasting serum samples on days 13, 15, 41 and 43

Plasma vitamin B6 and serum vitamin B12 and folic acid concentrations will be measured in fasting blood samples on days 13, 15, 41 and 43

~~Betaine, dimethylglycine and choline will be measured in fasting and non-fasting plasma samples collected at 6h after methionine loading at day 01, 15, 29, 43.~~

Blood lipids and liver enzymes will be analysed after each treatment period. The remaining blood parameters will be analysed after completion of the study.

15 Data management and statistics

15.1 Data management

The following SAS datasets will be prepared:
1. Pre-screening parameters
2. Body weight (days 01, 15, 29, 43)
3. Fasting homocysteine (days 01, 13, 15, 29, 41, 43)
4. Homocysteine after methionine (days 01, 15, 29, 43)
5. Blood lipids and liver enzymes (days 13, 15, 41 and 43)
6. Vitamin B6, B12 and folic acid (days 13, 15, 41 and 43)
7. ~~Betaine, dimethylglycine, choline (days 01, 15, 29 and 43)~~

For data set 1 raw data will partly be delivered on paper and entered using double data-entry and partly in electronic format. For data set 2 raw data will be delivered on paper and entered using double data-entry. For data set 3, 4, 5, 6 ~~and 7~~ raw data will be delivered in electronic format /on-line.

Raw data will be registered and will be converted to SAS data. All tranformations and changes in datasets will be controlled and registered. Locked datasets will be electronically transferred to the sponsor.

23 Approval of protocol amendment 02

23.1 Sponsor

P. Verhoef, PhD
Project Manager ____________________ ___-___-___
Signature Date (dd-mm-yy)

23.2 TNO Nutrition and Food Research

E.J. Brink, PhD ____________________ ___-___-___
Principal investigator Signature Date (dd-mm-yy)

W.A.A. Klöpping, MD, PhD ____________________ ___-___-___
Medical investigator Signature Date (dd-mm-yy)

C. Kistemaker, BSc ____________________ ___-___-___
Statistician Signature Date (dd-mm-yy)

A.F.M. Kardinaal, PhD ____________________ ___-___-___
TNO Management Signature Date (dd-mm-yy)

24 List of appendices

P4963 B07 : Distribution list
