# Supplementary material for: Effect of Homocysteine-Lowering Nutrients on Blood Lipids: Results from Four Randomised, Placebo-Controlled Studies in Healthy Humans
Source: PLoS Med. 2005 May 31;2(5):e135. doi: 10.1371/journal.pmed.0020135 (PMC1140947; doi:10.1371/journal.pmed.0020135)
Supplement: Table S5 — (25 KB DOC). [file pmed.0020135.st005.doc]

**Table S5. Non-serious adverse events per study^1^**.

| **Study** | **Total adverse events** | **Details adverse events^2^** |
| --- | --- | --- |
| Study 1 | 85 events in 27 subjects, reported according to WHO criteria and | 22 events were likely related to the methionine loadings^3^ in this study. These events included:   - Faintness (n=5 on placebo; n=3 on betaine; n=4 on folic acid) - Nausea (n=4 on placebo; n=1 on betaine; n=1 on folic acid) - Abdominal complaints (n=1 on placebo; n=1 on betaine) - Headache and tiredness (n=1 on betaine) - Blurred vision following methionine loading (n=1 on folic acid).   The remaining adverse events were diverse and unlikely related to the treatment. |
| Study 2 | 275 events in 70 subjects | 158 events were likely related to the methionine loadings^3^ in this study. These events included:   - Faintness (n=33 on placebo; n=22 on 1.5 g/d betaine; n=31 on 3 g/d betaine; n=46 on 6 g/d betaine) - Nausea/vomiting (n=8 on placebo; n=1 on 1.5 g/d betaine; n=5 on 3 g/d betaine; n=5 on 6 g/d betaine) - Straining of the neck during vomiting (n=1 on placebo) - Abdominal complaints (n=1 on 1.5 g/d betaine) - Headache and tiredness (n=1 on placebo; n=3 on 3 g/d betaine) - Sleeplessness (n=1 on placebo)   The remaining adverse events were diverse and unlikely related to the treatment. |
| Study 3 | 112 events in 31 subjects | The adverse events were diverse and unlikely related to the treatment. Most occurring events were headache/migraine (n= 59), and common cold/influenza (n=29). |
| Study 4 | 80 events in 21 subjects | 31 events were likely related to the methionine loadings^3^ in this study. These events included:   - Faintness (n=10 on placebo; n=13 on phosphatidylcholine) - Nausea/vomiting (n=1 on phosphatidylcholine) - Dyspepsia (n=2 on phosphatidylcholine) - Headache and tiredness (n=3 on phosphatidylcholine) - Palpitations (n=1 on placebo and n=1 on phosphatidylcholine)   Dyspepsia was reported 15 times during phosphatidylcholine treatment period, and once during placebo treatment.  The remaining adverse events were diverse and unlikely related to the treatment. |

^1^ Adverse events in studies 1, 2, and 4 are based on well-being questionnaires and spontaneous reporting. Adverse events in study 3 are based on spontaneous reporting.

^2^ n= number of events, one person can have more than 1 event.

^3^ Methionine loadings were done in studies 1, 2, and 4 to investigate effects of the treatments on plasma homocysteine (primary outcome) after methionine loading. Data on methionine loading are not reported in this paper.
